# Differential bumble bee gene expression associated with pathogen infection and pollen diet

**DOI:** 10.1186/s12864-023-09143-5

**Published:** 2023-03-29

**Authors:** Jonathan J. Giacomini, Lynn S. Adler, Benjamin J. Reading, Rebecca E. Irwin

**Affiliations:** 1 Department of Applied Ecology, North Carolina State University, Raleigh, NC 27695 USA; 2 Department of Biology, University of Massachusetts Amherst, Amherst, MA 01003 USA

**Keywords:** Sunflower pollen, Bumble bee, Parasitism, Immune transcripts, Gut epithelial cells, Detoxification

## Abstract

**Background:**

Diet and parasitism can have powerful effects on host gene expression. However, how specific dietary components affect host gene expression that could feed back to affect parasitism is relatively unexplored in many wild species. Recently, it was discovered that consumption of sunflower (*Helianthus annuus*) pollen reduced severity of gut protozoan pathogen *Crithidia bombi* infection in *Bombus impatiens* bumble bees. Despite the dramatic and consistent medicinal effect of sunflower pollen, very little is known about the mechanism(s) underlying this effect. However, sunflower pollen extract increases rather than suppresses *C. bombi* growth in vitro, suggesting that sunflower pollen reduces *C. bombi* infection indirectly via changes in the host. Here, we analyzed whole transcriptomes of *B. impatiens* workers to characterize the physiological response to sunflower pollen consumption and *C. bombi* infection to isolate the mechanisms underlying the medicinal effect. *B. impatiens* workers were inoculated with either *C. bombi* cells (infected) or a sham control (un-infected) and fed either sunflower or wildflower pollen ad libitum. Whole abdominal gene expression profiles were then sequenced with Illumina NextSeq 500 technology.

**Results:**

Among infected bees, sunflower pollen upregulated immune transcripts, including the anti-microbial peptide hymenoptaecin, Toll receptors and serine proteases. In both infected and un-infected bees, sunflower pollen upregulated putative detoxification transcripts and transcripts associated with the repair and maintenance of gut epithelial cells. Among wildflower-fed bees, infected bees downregulated immune transcripts associated with phagocytosis and the phenoloxidase cascade.

**Conclusions:**

Taken together, these results indicate dissimilar immune responses between sunflower- and wildflower-fed bumble bees infected with *C. bombi*, a response to physical damage to gut epithelial cells caused by sunflower pollen, and a strong detoxification response to sunflower pollen consumption. Identifying host responses that drive the medicinal effect of sunflower pollen in infected bumble bees may broaden our understanding of plant-pollinator interactions and provide opportunities for effective management of bee pathogens.

**Supplementary Information:**

The online version contains supplementary material available at 10.1186/s12864-023-09143-5.

## Background

Organisms are exposed to a wide range of environmental challenges, such as fluctuations in nutrient availability, ingestion of toxins, and exposure to pathogens. As a result, organisms modulate gene expression patterns at the transcriptional level to cope with these environmental challenges. Interactions between diet and pathogen infection can create feedbacks in gene expression that impact organism health. For example, in phytophagous insects, nutrient availability [1–4] or phytotoxins [5, 6] in the diet can reduce host immune gene expression, thus making a consumer more vulnerable to infection, or may enhance immune gene expression [7, 8], thus conveying health benefits to the consumer. Despite the large body of literature on multitrophic interactions [9–11], studies on whole genome transcriptomic responses to different diets and the interplay between diet and parasite infection remain rare. Here we focus on the relationship between bumble bees, pollen diet, and a protozoan pathogen to shed light on how specific dietary components affect host bee gene expression that could feed back to affect pathogen infection.

Consumption of sunflower (*Helianthus annuus*) pollen was recently discovered to reduce severity of the gut protozoan pathogen *Crithidia bombi* in *Bombus impatiens* worker and queen bumble bees by at least 80% relative to control pollen [12–14]. The medicinal effect of sunflower pollen may extend beyond bumble bees and *C. bombi*. For example, sunflower pollen reduced infection by the microsporidian *Nosema ceranae* in honey bees *Apis mellifera* [12, 15]. Further, Asteraceae pollen protected mason bees (*Osmia*) from brood parasitism [15]. Despite the dramatic and consistent medicinal effect of sunflower pollen on *C. bombi* infection in *B. impatiens*, we have yet to identify the mechanism(s) underlying this effect. However, since sunflower pollen extract increased rather than suppressed *C. bombi* growth in vitro [16], it is likely that sunflower pollen reduces *C. bombi* infection via changes in host physiological functions, such as immune and detoxification systems, or physical changes in the gut environment that prevent parasite growth and reproduction. There is an increasing interest in dietary ingredients that are appropriate to support insect pollinator health, including digestive and immune functions. Unfortunately, population declines have been observed for a number of bee species worldwide [17, 18] due to multiple stressors, including poor nutrition, habitat loss and pathogens [19, 20]. Thus, identifying mechanisms that underly the medicinal effect of sunflower pollen in infected bumble bees may broaden our understanding of pollinator disease ecology and provide opportunities to effectively manage bee pathogens.

Chemical or physical properties of sunflower pollen may have indirect negative effects on *C. bombi* mediated through changes in host bumble bee physiology. Sunflower pollen has relatively low protein content and lacks the essential amino acids methionine and tryptophan [21]. Many microbial gut parasites rely on their host for nutrition [22], and thus poor host nutrition can limit parasite growth and reproduction. However, the consumption of buckwheat pollen (*Fagopyrum escueluetum*), which matched sunflower pollen in crude protein and amino acid content, did not reduce *C. bombi* infection [12], and the consumption of a presumably nutritionally balanced sunflower pollen diet diluted with a diverse wildflower pollen blend (1:1 ratio by weight) significantly reduced *C. bombi* infection in bumble bees [23], ruling out poor host nutrition as the mechanism. Sunflower pollen also contains plant defensive compounds [24–26], saturated fatty acids and sterols that may have antimicrobial properties [27, 28]. Adler et al. [29] tested the effects of a variety of compounds found in sunflower pollen on *C. bombi* infection in bumble bees, including triscoumaroyl spermidine, rutin (a proxy for quercetin glycosides), and nine fatty acids, all of which failed to reduce *C. bombi* infection in vivo when mixed into non-medicinal control pollen diets. In a separate study, consumption of a sucrose solution spiked with chlorogenic acid reduced *Crithidia* sp*.* infection in bumble bees [30]. However, while Kostić et al. [24] detected chlorogenic acid in honey bee-collected sunflower pollen, a comprehensive study by our research group found no evidence of chlorogenic acid in sunflower pollen collected directly from flowers, but did find chlorogenic acid in petals and nectar [26]. Similar to what was found for sunflower pollen extracts, Palmer-Young et al. [31] demonstrated that chlorogenic acid did not have a direct toxic effect on *Crithidia* sp*.* cells, suggesting an indirect effect mediated through changes in host bumble bee physiology. Sunflower pollen also has a unique grain morphology characterized by the presence of conspicuous echinate spikes, which may provide a mechanical defence against over-exploitation by pollinivorous bees by decreasing digestibility [32], collectability [33] or by causing physical damage to the bee gut [34]. To date we are unaware of any study that has tested the effect of a spikey or rough material in the bee diet on gut-pathogen infection.

Analyzing changes in the bumble bee transcriptome in response to different diets and infection may shed light on the molecular pathways involved in the medicinal effect of sunflower pollen consumption*.* If sunflower pollen reduces infection via changes in the host bumble bee immune system, we would expect transcripts associated with canonical immune signaling pathways, including the Melanization and Encapsulation, Toll, Jak/STAT, IMD/JNK, or RNAi pathways [35], to be differentially expressed in sunflower pollen-fed bees compared to bees fed wildflower pollen control diet. In addition, a variety of detoxification genes are found in the genome of bumble bees, although to a lesser extent than other phytophagous insects [36]. Several studies have found that such genes play a major role in bee metabolism of phytotoxins and xenobiotics found in honey and pollen [37–40] and may also elicit an immune response in bees [41]. Congruent expression of putative immune and detoxification genes may indicate that plant defensive chemicals play an important role in the medicinal effect. Alternatively, if echinate sunflower pollen decreases digestibility, or causes physical damage to the bee gut, then we may expect changes in gene expression associated with plasma membrane repair that mediate active resealing of membrane disruptions to maintain homeostasis.

The objective of this study was to use a RNAseq-based whole transcriptome approach to identify key molecular pathways involved in the medicinal effect of sunflower pollen consumption in bumble bees infected with *C. bombi*. We analyzed differences in gene expression profiles of adult *B. impatiens* workers inoculated with live *C. bombi* cells or a sham control, and then either fed a sunflower or wildflower pollen diet. Using a combination of traditional frequentist statistics and machine learning techniques, we found that consumption of sunflower pollen enhances bumble bee immune response to *C. bombi*, stimulates detoxification processes and upregulates genes associated with physical damage to or remodeling of gut epithelial cells. The data generated in this study provide a strong foundation to further explore the chemical and structural properties of sunflower pollen that drive the medicinal effect in bumble bees.

## Results

**Fig. 1:**
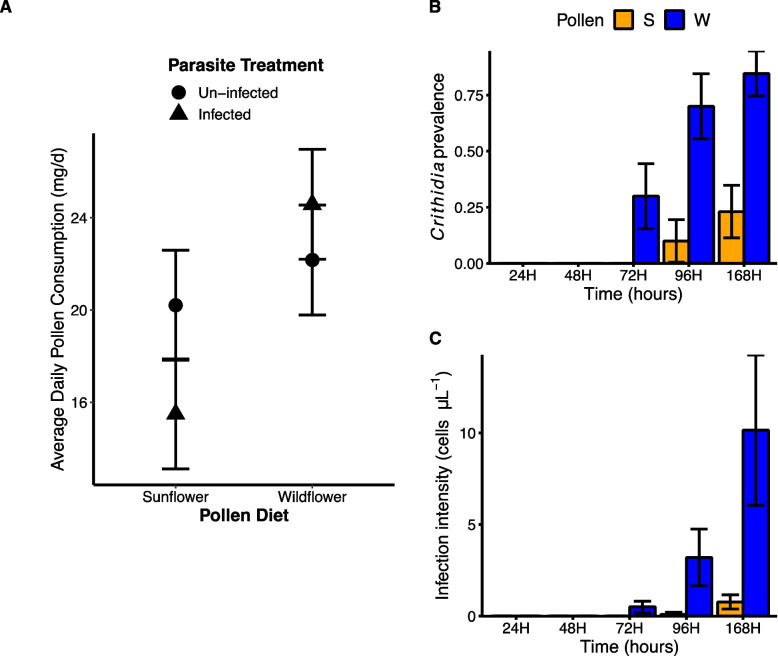
(**A**) Average daily rate of sunflower or wildflower pollen consumption by *Bombus impatiens* workers either un-infected (circles) or infected with *Crithidia bombi* (triangles) and submitted for RNA sequencing. Points represent model adjusted means and errors bars 1 SE ± of the mean. These results were used to inform methods for the RNA seq experiment. Only bees that consumed a net positive amount of pollen throughout the 72-h post-inoculation period were selected for RNA sequencing. (**B** and **C**) Mean proportion *Bombus impatiens* workers with detectable *Crithidia bombi* infection (**B**) and infection intensity (**C**). Bees were provided either sunflower pollen (S; orange bars) or control wildflower pollen (W; blue bars) for a period of 24, 48, 72, 96 or 168 h (H) post-inoculation. Bars and error bars represent model adjusted means and one standard error, respectively

*Inoculation efficacy and pollen consumption.* Only a subset of the initial group of bees were chosen for RNA sequencing (see *Methods: Inoculation treatment*). The remainder (hereafter termed non-RNAseq bees) were reserved to determine infection prevalence and intensity under the treatment conditions to assess inoculation efficacy. All but one (out of 18) of the non-RNAseq bees fed wildflower pollen were infected with *C. bombi*, suggesting successful inoculation for the RNAseq bees. Infected wildflower-fed bees had an average *C. bombi* intensity of 33.44 ± 12.3 cells/0.02 μL (mean ± SE), which is an approximately 42-fold change increase compared to the initial inoculum. Sunflower pollen significantly reduced the prevalence of *C. bombi* infection by 35.07% (χ^2^ = 10.678, *p* = 0.001) and the intensity of infection by 85.77% (χ^2^ = 5.866, *p* = 0.015) relative to wildflower pollen. The interaction between pollen diet and the average daily rate of pollen consumption, as well as the main effect of average daily rate of pollen consumption, did not have a significant effect on *Crithidia* infection intensity or prevalence (χ^2^ < 0.388, *p* > 0.226, for both) in the non-RNAseq bees. Of the RNAseq bees, sunflower-fed bees had 24% lower average rate of pollen consumption compared to wildflower-fed bees (F_1,16_ = 5.374, p = 0.034; Fig. 1A).

### Assembly and blast

In total, between 13,702,311 and 60,011,808 cleaned reads were obtained after sequencing and trimming (Table S1, *Supporting Information*). The average mapping rate of clean reads to the *Bombus impatiens* genome was 91.38 ± 1.27%, resulting in 22,726 unique transcripts. Reannotating the transcripts using OmicsBox blastx using the nr database against all arthropods yielded a total of 17,077 hits. The greatest number of top BLAST hits were found in *B. impatiens*, with the top five from *Bombus* (Figure S1, *Supporting Information*), giving us confidence in our read quality.

### Differential gene expression—Infected bees: sunflower vs. wildflower

**Fig. 2:**
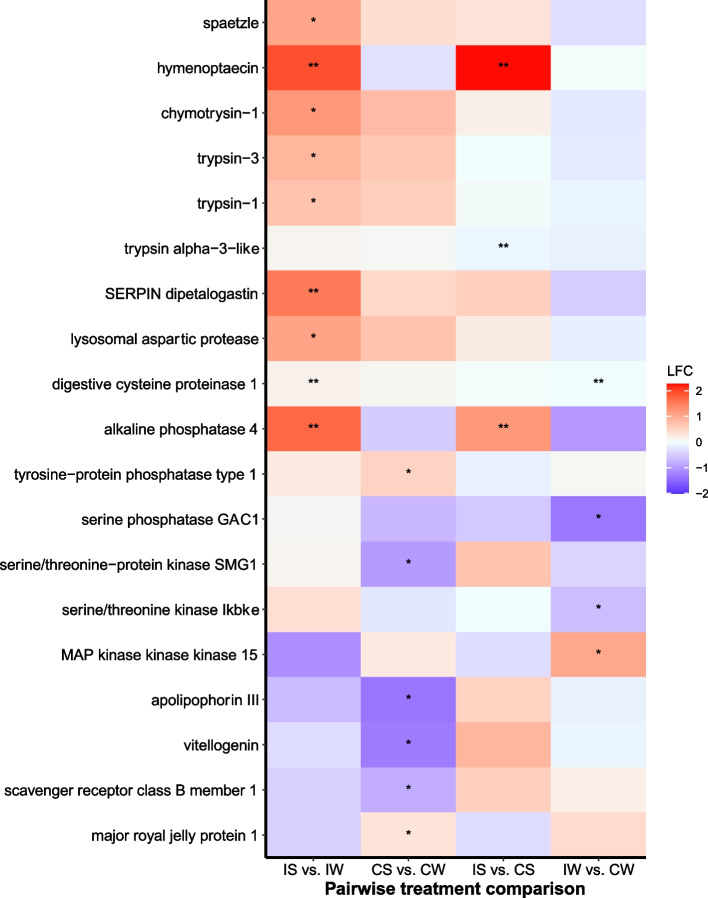
Gene expression profiles of putative immune transcripts in *Bombus impatiens* workers inoculated with the gut protozoan parasite *Crithidia bombi* (I) or inoculated with a sham control (C) and fed either sunflower (S) or wildflower (W) pollen. Colors indicate shrunken log fold changes (LFC) estimated using a negative binomial model. Double asterisks indicate differentially expressed transcripts based on negative binomial DESeq2 model and FDR < 0.05. Single asterisk indicates important differentially expressed transcripts based on machine learning analysis

**Fig. 3:**
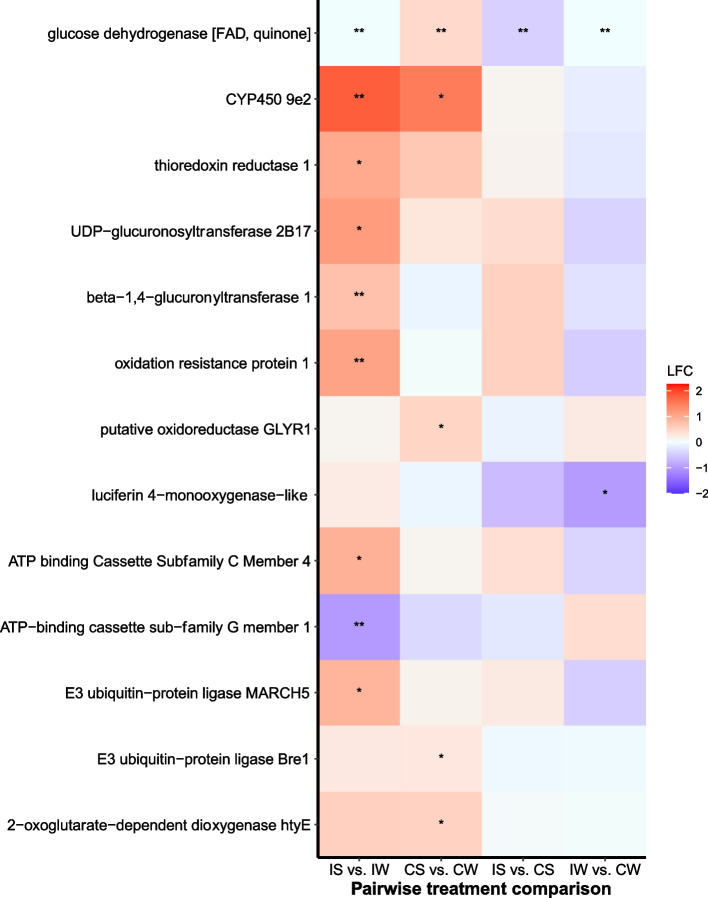
Gene expression profiles of putative detoxification transcripts in *Bombus impatiens* workers inoculated with the gut protozoan parasite *Crithidia bombi* (I) or inoculated with a sham control (C) and fed either sunflower (S) or wildflower (W) pollen. Colors and symbols as in Fig. 2

**Fig. 4:**
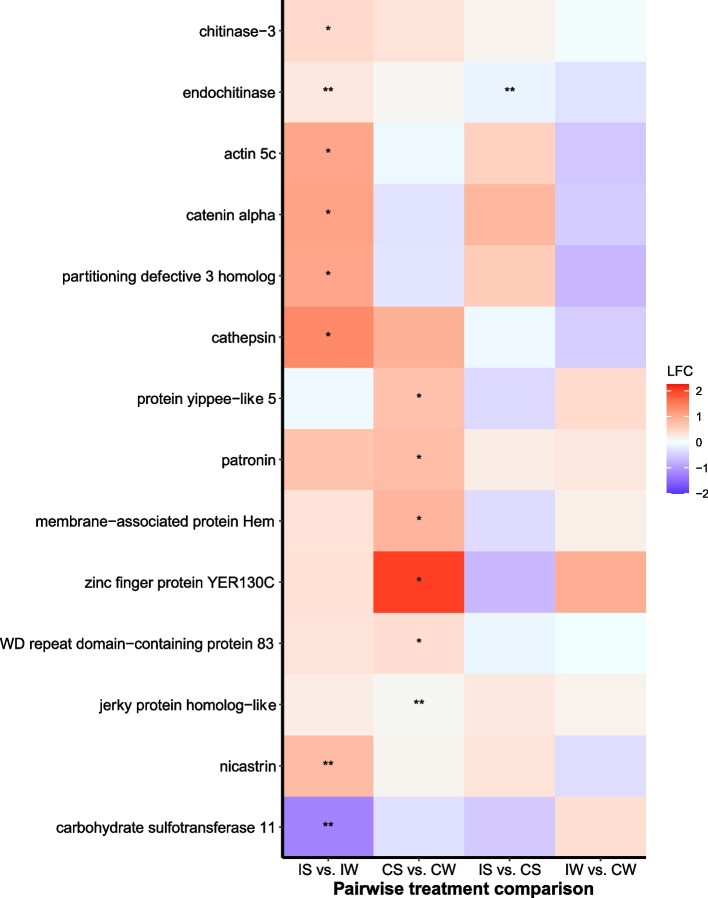
Gene expression profiles of gut morphology transcripts in *Bombus impatiens* workers inoculated with the gut protozoan parasite *Crithidia bombi* (I) or inoculated with a sham control (C) and fed either sunflower (S) or wildflower (W) pollen. Colors and symbols as in Fig. 2

Among infected bees*,* 40 transcripts were differentially expressed between sunflower- and wildflower-fed bees based on the DESeq2 model (FDR < 0.05; Table S2, *Supporting Information*). Notably, four transcripts associated with the innate immune system were significantly upregulated in infected sunflower-fed bees (Fig. 2), including the anti-microbial peptide *hymenoptaecin* (XP_003494933)*,* a *serine protease inhibitor dipetalogastin* (XP_012236217), a plasma membrane-bound glycoprotein *alkaline phosphatase 4-like* (XP_012241779), and *digestive cysteine proteinase 1* (XP_003494144). Additionally, four transcripts associated with detoxification and oxidative stress were upregulated in infected sunflower-fed bees (Fig. 3), including *glucose dehydrogenase [FAD, quinone]-like* (XP_012248181), *cytochrome P450 9e2-like* (XP_033174299) *oxidation resistance protein 1 isoform X6* (XP_024222768) and *beta − 1,4 − glucuronyltransferase 1* (XP_003491810). Two transcripts associated with gut morphology were upregulated in sunflower-fed bees (Fig. 4), including the glycoside hydrolase *endochitinase* (XP_012241960) and the gamma secretase *nicastrin* (XP_012247186).

Based on machine learning, we were able to correctly classify sunflower-fed from wildflower-fed infected bumble bees in 100% (SD = 0%) of the instances when using the top 160 through 105 ranked transcripts (Figure S2, *Supporting Information*). Re-training the SMO model with the randomized data sets, the overall mean percent correct classification was 47.31% (SD = 24.09%), the average kappa statistic was -0.04 (SD = 0.46) and the AUROC was 0.48 (SD = 0.24), indicating that true learning occurred in the optimized SMO model with 160 top ranked transcripts. The IDs, gene functions and expression levels of the top 160 ranked transcripts are presented in Table S3 (*Supporting Information*).

Notably, machine learning identified importance of a number of transcripts associated with the innate immune system that were upregulated in infected sunflower-fed bees compared to infected wildflower-fed bees (Fig. 2), including Toll pathway receptor *spaetzle 4* (XP_033361233), several serine proteases, including *transmembrane protease serine 9-like* (XP_033180356)*, serine protease inhibitor dipetalogastin* (XP_012236217) and *probable serine/threonine-protein kinase samkC* (XP_012245395), *chymotrypsin-1* (XP_003485243)*, trypsin-3* (XP_012240481) and *trypsin-1* (XP_012240481). Machine learning also identified several transcripts associated with detoxification that were upregulated in infected sunflower-fed bees (Fig. 3), including *UDP-glucuronosyltransferase 2B17-like* (XP_033176691)*,* three transcripts for *cytochrome P450 9e2-like* (XP_033174299 and XP_003484581)*,* two transcripts for *oxidation resistance protein 1* (XP_024222768)*, thioredoxin reductase 1* (XP_012247756) and *E3 ubiquitin-protein ligase MARCH5* (XP_003492433)*.* A transcript for *probable cytochrome P450 305a1* (XP_003484727) was downregulated in infected sunflower-fed bees. Six transcripts associated with gut morphology, epithelium repair and maintenance were also identified by machine learning, which were upregulated in infected sunflower-fed bees (Fig. 4), including two transcripts for *chitinase-3-like protein 1* (XP_003488774), *actin 5c* (XP_014484761), *catenin alpha* (XP_017011441), *Partitioning defective 3 homolog* (XP_012236781), and two transcripts for *lysosomal aspartic protease* (XP_003489428).

### Functional enrichment—infected bees: sunflower vs. wildflower

We did not find significant enrichment of any Gene Ontology (GO) biological process or molecular function terms for DEGs from the DESeq2 model. However, GO enrichment analysis based on the combination of the top 160 ranked transcripts identified by machine learning indicated significant enrichment of proteolysis, glucosidase, hydrolase, carboxypeptidase, exopeptidase and peptidase activities, as well as several carbohydrate metabolic processes in infected sunflower-fed bees compared to infected wildflower-fed bees. (Figure S3, *Supporting Information*), suggesting a metabolic response to xenobiotics and pollen nutrients (i.e., proteins, lipids and starches).

#### IPA: canonical pathways

We used Qiagen Ingenuity Pathway Analysis (IPA) software to further interpret functions of differentially expressed transcripts in each treatment pairwise comparison. A total of 104 out of the 160 top ranked optimal transcripts identified by machine learning were successfully mapped into IPA; 45 transcripts were uncharacterized, and we were unable to find human, rat or mouse orthologs for 11 transcripts. The top enriched canonical pathway was NRF2-mediated oxidative stress response (Table S4; *Supporting Information*), which was predicted to be activated in infected sunflower-fed bees compared to infected wildflower-fed bees (z-score = 2.00, *p*-value < 0.0001). This pathway elicits a cellular defense response to oxidative stress, including induction of detoxifying enzymes and antioxidant enzymes. Several other canonical pathways that overlap with the NRF2-mediated oxidative stress response pathway and play a role in response to oxidative stress were also enriched, including the thioredoxin pathway, acetone degradation, nicotine degradation II & III pathways, several melatonin degradation pathways, the epithelial adherens junction signaling pathway, and the aryl hydrocarbon receptor signaling pathway. The epithelial adherens junction signaling pathway also plays an important role in the maintenance of epithelial cell layers.

### Differential gene expression—uninfected bees: sunflower vs. wildflower

Among uninfected bees, 10 transcripts were differentially expressed between sunflower- and wildflower-fed bees based on the DESeq2 model (FDR < 0.05; Table S5, *Supporting Information*). Seven transcripts were upregulated in uninfected sunflower bees, four of which were characterized: *dehydrogenase [FAD, quinone]-like* (XP_033180074), *jerky protein homolog-like* (XP_012248162)*, RNA-directed DNA polymerase from mobile element jockey-like* (XP_012244110) and the *putative odorant receptor 92a* (XP_024226497)*.* Three transcripts were down-regulated in uninfected sunflower bees, one of which was characterized: *dynein beta chain, ciliary-like* (XP_033357315).

Based on machine learning, we were able to correctly classify uninfected sunflower-fed from wildflower-fed bumble bees in 100% of the instances with the top 141 through 114 ranked transcripts (Figure S4, *Supporting Information*). Re-training the SMO model with the randomized data sets, the overall mean percent correct classification was 51.28% (SD = 21.54%), the average kappa statistic was 0.03 (SD = 0.44) and the AUROC was 0.51 (SD = 0.22), indicating that true learning occurred in the optimized SMO model with 141 top ranked transcripts. The IDs, gene functions and expression levels of the top 141 ranked transcripts are presented in Table S6 (*Supporting Information*).

Similar to gene expression patterns for infected bees, machine learning identified transcripts associated with the immune system (Fig. 2), detoxification (Fig. 3) and gut morphology (Fig. 4) as important for distinguishing between sunflower and wildflower bees. Notably, sunflower pollen upregulated the pro-inflammatory regulator *tyrosine-protein phosphatase* (XP_012236351) and *major royal jelly protein 1* (XP_012247599), the latter of which has been shown to have antimicrobial effects in bees [42] and upregulated in response to *Crithidia* sp. infection [43]. In addition, sunflower bees upregulated *WD repeat domain-containing protein 83* (XP_012239808), a scaffold protein that regulates the Extracellular Signal Related Kinase (ERK) cascade associated with an inflammatory response to wounding [44] and enterocyte gut epithelial cell proliferation [45].

### Functional enrichment—uninfected bees: sunflower vs. wildflower

We did not find significant enrichment of any GO biological process or molecular function terms for DEGs identified in the DESeq2 model. However, GO enrichment analysis based on the combination of the 141 top ranked transcripts identified by machine learning indicated that a number of biological processes involving pigmentation and oxidation–reduction (redox) reactions were significantly enriched (Figure S5, *Supporting Information*), indicating a detoxification response in uninfected sunflower-fed bees.

#### IPA: canonical pathways

A total of 68 out of the 141 top ranked optimal transcripts identified by machine learning were successfully mapped into IPA; 58 transcripts were uncharacterized, and we were unable to identify human, rat or mouse orthologs for 15 transcripts. Consistent with the IPA analysis for infected bees, a number of significant canonical pathways associated with xenobiotic metabolism, and gastrointestinal physiology were enriched in uninfected bees fed sunflower pollen. The top enriched canonical pathway was epithelial adherens junction signaling pathway (Table S4, *Supporting Information*), followed by sorbitol degradation, stearate biosynthesis, calcium signaling, and protein kinase A signaling. The NRF2-mediated oxidative stress response and the LPS/IL-1 Mediated Inhibition of RXR pathways were also enriched, both of which were predicted to be activated in infected sunflower-fed bees compared to infected wildflower-fed bees. IPA was unable to predict activation of any of the enriched canonical pathways in uninfected sunflower-fed bees.

### Differential gene expression—sunflower-fed bees: infected vs. uninfected

Among sunflower-fed bees, 12 transcripts were differentially expressed between infected and uninfected bees based on the DESeq2 model (FDR < 0.05; Table S7, *Supporting Information*). Of immune transcripts, the antimicrobial peptide *hymenoptaecin* (XP_003494933), the plasma membrane-bound glycoprotein *alkaline phosphatase 4* (XP_012241779), and the proteolytic enzyme *trypsin alpha-3-like* (XP_003491285) were upregulated in infected bees (Fig. 2). A transcript for a detoxification enzyme *glucose dehydrogenase [FAD, quinone]-like* (XP_033180074) was downregulated in infected bees (Fig. 3). The glycoside hydrolase *endochitinas*e (XP_012241960) was significantly upregulated in infected sunflower-fed bees (Fig. 4).

Machine learning fairly predicted infection treatment among sunflower-fed bees (Figure S6, *Supporting Information*), in most instances only reaching 80% correct classification with considerably large standard deviation (> 30%). The best machine learning classification was obtained from the top 80 through 78 ranked transcripts (% CC: 90.00 ± 30.02; mean ± SD); IDs and gene functions are presented in Table S6 (*Supporting Information*). Re-training the SMO model with the randomized data sets, the overall mean percent correct classification was 47.65% (SD = 20.06%), the average kappa statistic was -0.04 (SD = 0.36) and the AUROC was 0.48 (SD = 0.20). Substantial overlap between variation (SD) around the average percent correct classification between the negative control and optimized model, indicates that true learning failed in the SMO model with 80 top ranked transcripts.

### Functional enrichment—sunflower-fed bees: infected vs. uninfected

We did not find significant enrichment of any GO biological process or molecular function terms for DEGs identified in either the DESeq2 model or the top 80 ranked transcripts identified by machine learning. Since machine learning poorly classified infection status among sunflower-fed bees, and so few transcripts were differentially expressed in the DESeq2 model, we did not perform IPA analysis to avoid misleading results.

### Differential gene expression—wildflower-fed bees: infected vs. uninfected

Among wildflower-fed bees, 17 transcripts were differentially expressed between infected and uninfected bees based on the DESeq2 model (FDR < 0.05; Table S9, *Supporting Information*). Notably, the proteolytic enzyme *digestive cysteine proteinase 1* (XP_003494144) and the detoxification enzyme *glucose dehydrogenase [FAD, quinone]-like* (XP_012248181) were downregulated in infected bees (Fig. 2). No transcripts associated with gut morphology were differentially expressed between infected and uninfected wildflower-fed bees (Fig. 4).

The best machine learning classification was obtained from the top 98 through 41 ranked transcripts (% CC: 100.00 ± 00.00; mean ± SD; Figure S7, *Supporting Information*); the IDs, gene functions and expression levels are presented in Table S10 (*Supporting Information*). Re-training the SMO model with the randomized data sets, the overall mean percent correct classification was 47.70% (SD = 20.13%), the average kappa statistic was -0.03 (SD = 0.41) and the AUROC was 0.48 (SD = 0.22), indicating that true learning occurred in the optimized SMO model with 98 top ranked transcripts.

Notably, machine learning identified importance of several transcripts associated with an immune response that were downregulated in infected wildflower bees (Fig. 2), including a serine/threonine kinase *inhibitor of nuclear factor kappa-B kinase subunit epsilon* (XP_003486634) and *serine/threonine-protein phosphatase 1 regulatory subunit GAC1-like* (XP_033179143). Machine learning also identified *luciferin 4-monooxygenase-like isoform X6* (XP_003491563), which was downregulated in infected bees (Fig. 4) and in previous work is associated with detoxification in honey bees [39]. No transcripts associated with gut morphology were identified by machine learning as important for distinguishing infected from uninfected wildflower bees.

### Functional enrichment—wildflower-fed bees: infected vs. uninfected

We did not find significant enrichment of any GO biological process or molecular function terms for either the DEGs identified in the DESeq2 model or the top 98 ranked transcripts identified by machine learning.

#### IPA: canonical pathways

A total of 39 out of the 98 top ranked optimal transcripts identified by machine learning were successfully mapped into IPA; 56 transcripts were uncharacterized, and we were unable to identify human, rat or mouse orthologs for 3 transcripts. Six canonical pathways were enriched in infected compared to uninfected wildflower-fed bees (Table S4, *Supporting Information*). The top enriched canonical pathway was Choline Degradation I, driven solely by large downregulation of choline dehydrogenase (*Chdh*) in infected bees. Several nucleotide metabolism pathways were enriched; upregulation of phosphoribosyl pyrophosphate synthetase 1 (*Prps1*) was associated with enrichment of the PRPP Biosynthesis I pathway, and downregulation of acid phosphatase 3 (*Acp3*) was associated with enrichment of the NAD Phosphorylation and Dephosphorylation, Urate Biosynthesis/Inosine 5-phosphate degradation, Guanosine Nucleotides Degradation III and Adenosine Nucleotides Degradation II pathways.

## Discussion

Consuming sunflower pollen resulted in the upregulation of transcripts associated with multiple physiological processes. Among infected bees, sunflower pollen upregulated transcripts associated with the Toll-mediated innate immune system, putative detoxification transcripts and transcripts associated with the repair and maintenance of gut epithelial cells. Among uninfected bees, sunflower pollen upregulated similar detoxification transcripts and transcripts associated with repair and maintenance of gut epithelial cells, but not a Toll-mediated immune response. In uninfected wildflower-fed bees, we did not detect upregulation of the same Toll-mediated immune response or detoxification transcripts in infected bees, but instead found signs of immune deactivation. Taken together, these results suggest that consuming sunflower pollen causes a different immune response than consuming wildflower pollen as well as a detoxification response. Moreover, gene expression patterns suggest that sunflower pollen may cause remodeling of or damage to the gut lining; such changes to the gut lining have the potential to feedback and enhance an effective immune response to *C. bombi* infection. Taken together, we show that there was not an overwhelming signal for a single specific mechanism, but instead several complex and non-mutually exclusive mechanisms (i.e., immune, detox, and/or gut morphology) that may drive the medicinal effect of sunflower pollen on *Crithidia* infection in host bumble bees. Further experimental research supported by RT-PCR validation is needed to disentangle the chemical and mechanical effects of sunflower pollen on bee gene expression. Our study provides important analyses of transcriptomic data that can set the stage for many future experiments that may provide functional insight into the mechanisms underlying the medicinal effects of sunflower pollen, as well as broaden our understanding of how diet influences disease ecology.

One major hypothesis for the mechanism underlying the medicinal effect of sunflower pollen is that detoxification enhances the immune system. We found concurrent upregulation of both a Toll-mediated immune response and several putative detoxification enzymes in infected sunflower-fed bees, but not in uninfected sunflower-fed bees or wildflower-fed bees regardless of infection treatment. In insects, P450 cytochrome enzymes (CYPs) are known to regulate both pathogen infection, by producing reactive oxygen radicals (e.g., nitric oxide), and detoxification of xenobiotics [46]. CYP variants play a role in resistance to fungal infection in silkworms [47], varroa mite resistance in honey bees [48], metabolism of potent insecticides in bumble bees [49], and *Crithidia* infection in bumble bees [43]. In honey bees, the secondary metabolites found in pollen and pesticides upregulate both detoxification and immunity transcripts, including several CYPs and the AMPs *abaecin* [39] and *hymenoptaecin* [50, 51], which enhanced the immune response of honeybees against both a microsporidian pathogen and viral infections [50]. Oxidoreductases, including FAD-GLD that was upregulated in sunflower-fed bees, also play a major role in insect detoxification and act as a messenger to induce immune-related transcripts [52]. Interestingly, Cox-Foster and Stehr [53] suggested that FAD-GLDs interact with phenoloxidase, and play an important role in the killing mechanism of pathogens by reducing quinone, which leads to the production of superoxide radicals that create a toxic environment for pathogens. Further research is needed to determine if detoxification of phytochemicals in sunflower pollen is independent of the immune response to *C. bombi* infection in bumble bees.

A major challenge for living organisms is to maintain homeostasis in the face of multiple internal and external stressors, such as pathogen infection, variation in nutrient supply and exposure to toxins. In response, complex immune systems have evolved to eliminate the potential threat and re-establish homeostasis without causing excessive damage to healthy cells and tissues. We found signs of deactivation of an immune response in infected wildflower-fed bees 72 h post-inoculation, including downregulation of functional communication along the gut-brain axis, serine kinases, serine protein phosphatases, acid phosphatase and a cysteine proteinase. This pattern agrees with another study that found temporal expression of immune transcripts associated with Toll and melanization immune pathways in insects exposed to pathogens, reducing expression at 72 h post-inoculation [54]. Downregulation of an immune response may indicate that infection has bypassed the host’s first line of defense and reflect diverting energy to other components of host physiology. This may prevent the toxic accumulation of reactive oxygen species and the production of energetically costly immune effectors, such as AMPs. Consequently, temporal variation in immune responses may obscure the underlying mechanism of medicinal sunflower pollen. This is supported by a study from Locascio et al. [55] that found consuming sunflower pollen for the first 3.5 days or all 7 days after being inoculated with *Crithidia*, but not 3.5 days after inoculation, reduced cell counts in bees compared to those fed a negative control pollen. This suggests an important relationship between the timing of sunflower pollen consumption and the establishment of *Crithidia* infection in host bumble bees. We thus propose a 24 h time course experiment ranging from 3–4 days before inoculation to 3–4 days after inoculation may shed light on whether duration and timing of exposure to sunflower pollen are important factors that modulate host bumble bee gene expression.

In addition to digestion and nutrient absorption, the digestive tract plays an important role in protecting an organism from absorption of ingested xenobiotics that cause oxidative stress, such as plant defense compounds or pesticides. Sunflower pollen consumption strongly upregulated multiple transcripts that are involved in the primary metabolism of xenobiotics, including a P450 cytochrome (CYP), a quinone oxidoreductase, two glucuronosyltransferases, an ATP-binding cassette (ABC) transporter, thioredoxin reductase and E3 ubiquitinating proteins. Although our research group has tested the effect of several sunflower pollen compounds on infection, we have not yet identified any that reduce *Crithidia* infection in bumble bees [29]. Pesticides are commonly used on sunflower crops to suppress weeds, herbivorous insects and plant pathogens [56] and can pose a substantial risk for bees [57]. The upregulation of multiple detoxification enzymes in sunflower-fed bees could be an indication of pesticide contamination in sunflower pollen. However, while the pesticide residues in pollen used in this study were not measured, both the sunflower and wildflower pollen were sourced from the same suppliers as in Giacomini et al. [12], which did measure pollen pesticide levels. In that study, a greater diversity of pesticide residues was found in wildflower compared to sunflower pollen, all but two of which were at trace levels. The two that were above trace levels were both miticides used to treat varroa mites in honey bee colonies. Sunflower pollen also contained a different miticide used to treat varroa in honey bees. Given that pesticide levels were low overall and greater in wildflower than sunflower pollen, it seems unlikely that pesticides are responsible for upregulation of detoxification transcripts in sunflower-fed bees.

In addition to detoxification and an immune response, gene expression patterns indicated wound healing activity in the abdominal gut tissues of *B. impatiens* in response to consuming sunflower pollen, including the enrichment of the Epithelial Adherens Junction Signaling pathway (EAJS), as well as transcripts associated with the formation of cellular surface protrusions, proliferation of fibroblasts and activation of signaling pathways in the brush border membrane. Wound healing in the digestive tract is a dynamic process that requires coordination between the proliferation of new cells, the reorganization of intracellular matrices and both inter- and intra-cellular signaling pathways that facilitate cell-to-cell adhesion [58]. The cells of the epithelial layer are joined together by tight junctions composed of a branching network of transmembrane proteins, thus forming a contiguous and relatively impermeable membrane. Adherens junctions are specialized intercellular junctions, in which actin filaments are linked to cadherin molecules of adjacent cells via catenin molecules. These junctions perform multiple functions, including initiation and stabilization of cell-to-cell adhesion [59, 60]. Disruption of epithelial cells by sunflower pollen may thus trigger a wound healing response or remodeling of the gut in bumble bees that involves the reformation of intercellular junctions. Since *Crithidia* sp*.* require tight adhesion to the gut lining in bumble bees to establish infection [61], it is plausible that phytosterols in sunflower pollen or echinate spines, which are a particularly notable trait of sunflower pollen morphology [62], cause damage to the gut lining, which in turn prevents adhesion and reduces proliferation of *Crithidia* sp.

While this study is unable to differentiate between chemically- or mechanically-induced damage caused by sunflower pollen consumption, upregulation of both a detoxification response and a response to wound healing is consistent with recent evidence that closely related *Taraxacum* pollen damaged the gut lining of *B. terrestris* bumble bees [34]. Similarly, that study was unable to differentiate between a chemical or mechanical cause of damage since a non-*Taraxacum* pollen diet spiked with phytosterols found in *Taraxacum* pollen and crushed *Taraxacum* pollen both induced damage to the gut lining of the digestive tract. On one hand, crushed pollen could increase abrasiveness and cause mechanical damage to the gut lining. Alternatively, crushed pollen could release phytochemicals that would be otherwise trapped in undigested pollen grains, and thus increase exposure to toxins that damage the gut lining. In our study, both sunflower pollen and the control wildflower pollen diets were provided to bees in the form of a paste, which required mechanical breakdown of honey-bee collected pollen pellets before adding water. During that process a small proportion of pollen grains are indeed fractured (JJG, *personal observation*), but since both diets were treated the same, we can rule out mechanical damage to the gut lining caused by fragmented pollen grains. However, *Helianthus sp*. pollen grains are much more echinate than *Taraxacum sp.* pollen, so we cannot rule out mechanical damage caused by intact pollen grains. If abrasiveness of echinate pollen causes damage to the gut lining, then pollen diets that contain a high proportion of pollen species with echinacious spines, regardless of plant family, will cause damage.

In insects, the peritrophic membrane (PM) is regularly shed and replaced via a well-regulated synthesis and turnover of chitin [63] to facilitate both growth and morphogenesis. The PM effectively functions to filter small molecules and aid in nutrient absorption, as well as protect the gut epithelium from damage from abrasive foods or pathogen invasion [34, 43, 64, 65]. Infected bumble bees fed sunflower pollen increased expression of *chitinase-3-like protein 1* compared to wildflower-fed bees and increased expression of *endochitinase* compared to uninfected sunflower-fed bees. Differential expression of host bumble bee chitinases in response to *Crithidia* infection has been demonstrated in previous work [43], but an effect on *Crithidia* infection has not been detected. One hypothesis is that host bumble bees respond to *C. bombi* infection by increasing the turnover of the peritrophic membrane (PM) that lines the insect midgut, which physically removes *C. bombi* cells from the digestive tract. However, we did not see upregulation of PM-associated transcripts in infected wildflower-fed bees, indicating a synergistic interaction between sunflower pollen consumption and *C. bombi* infection, possibly mediated by abrasive damage to the PM by sunflower pollen. If the combination of sunflower pollen consumption and *C. bombi* infection increases the turnover of PM, then we may expect differences in the amount of chitin in the bumble bee peritrophic membrane, which can be quantified by image processing [66].

## Conclusions

The data generated from this study provide an important foundation to disentangle the mechanism(s) underlying the medicinal effect of sunflower pollen in bumble bees. Chemical or mechanical properties of sunflower pollen may enhance the bumble bee immune system, facilitating targeted destruction of *C. bombi* cells. Detoxification of phytotoxins found in sunflower pollen may generate a toxic environment for *C. bombi* cells, or may stimulate and enhance a host immune response. Similarly, echinate sunflower pollen or phytosterols may cause damage to the gut lining, directly preventing growth and reproduction of *C. bombi*, or stimulating an effective host immune response. Future research should focus on disentangling the effects of chemical and physical properties of sunflower pollen on host bumble bee physiology, and the implications for *C. bombi* infection. Identifying plant traits and host physiological responses that drive the medicinal effect of sunflower pollen in infected bumble bees may broaden our understanding of pollinator disease ecology and provide opportunities for effective management of bee pathogens.

## Methods

### Study system

*Bombus impatiens* is a native eusocial bee species in North America, ranging from Maine to Ontario to the eastern Rocky Mountains and south through Florida [67]. They are generalists that visit a range of agricultural and native plants. *B. impatiens* have also been domesticated for crop pollination services throughout much of North America [68–70], subsequently making them a widely utilized study species. An annotated reference genome for *B. impatiens* [71] is available from the National Center for Biotechnology Information (NCBI). At the time of this study, NCBI BIMP_2.2 (GenBank assembly accession: GCA_000188095.4) contained 13,161 transcripts that code for 24,471 proteins.

*Crithidia bombi* (Zoomastigophora: Trypanosomatidae) is an infectious protozoan gut pathogen that can be contracted at flowers via fecal transmission and can also be horizontally transmitted within colonies [72, 73]. *Crithidia* sp. reduce learning and foraging efficiency in worker bumble bees [74, 75], slow colony growth rates, especially early in the colony life cycle [76], reduce the likelihood of successful reproduction in wild colonies [77], and reduce infected queen fitness [78]. *Crithidia* sp. infection is common; for example, *Crithidia* sp. infected over 60% of wild-caught *B. impatiens* in western MA [79] and commercial colonies can have high levels of infection [74].

Sunflowers (*Helianthus* sp.) belong to a large and diverse family (Asteraceae) with over 32,000 described species [80]. *Helianthus annuus* is a major domesticated oilseed crop cultivated worldwide and a native US wildflower [81]. With nearly two million acres of sunflowers planted in the US [82] and ten million acres planted in Europe annually [83], the high abundance of cultivated sunflowers combined with large nectar and pollen yields make it an important resource for bees.

### Preparing inoculation treatments

Live *Crithidia bombi* cells were harvested from three wild *B. impatiens* workers collected near Stone Soup Farm, Hadley, MA, USA in 2014 (42.363911 N, -72.567747 W) and housed in commercial colonies of *B. impatiens* thereafter. The *Crithidia* species was identified in a previous study and confirmed to be *C. bombi* [84]. Both the *C. bombi* source colony and experimental colony used in this experiment were purchased from Koppert Biological Systems (Howell, MI, USA). Colonies were fed with 30% sucrose solution and mixed wildflower pollen throughout their lifetimes and housed in a dark room at 21 – 24ºC and ~ 50% rh. We made *C. bombi* inoculum using an established protocol [12, 85, 86]. Briefly, bee digestive tracts of 15 workers, excluding the honey crop, were removed with forceps, placed into 1.5 mL microcentrifuge tubes with 300 μL of distilled water, and ground with a pestle. We allowed each sample to rest at room temperature for 4–5 h so that gut material settled and *C. bombi* cells could ascend into the supernatant. *Crithidia bombi* cells were counted from a 0.02 μL sample of supernatant per bee with a Neubauer hemacytometer under a compound light microscope at 400X magnification. We then mixed 150 μL of the supernatant with distilled water to achieve a concentration of 2400 cells μL^−1^. The sample was then mixed with an equal volume of 50% sucrose solution to yield inoculum with 1200 cells μL^−1^ in 25% sucrose. We made the sham inoculum following the same procedure as the *C. bombi* inoculum, but instead used the digestive tracts of five bees from the un-infected experimental colony.

### Preparing pollen diets

We prepared two pollen diet treatments – sunflower and wildflower. Honey bee-collected sunflower pollen pellets were obtained from Changge Hauding Wax Industry (China) and sorted by color to remove impurities. We verified a pure batch of sunflower pollen by staining five samples with basic fuschin dye [87] and visually confirming only sunflower pollen was present with a compound microscope at 400X magnification. Honey bee-collected mixed wildflower pollen pellets were obtained from Koppert Biological Systems (Howell, MI, USA) and microscopically confirmed to contain < 5% Asteraceae pollen, identified by having spines on the exine [62]. Experimental pollen diets were provided to bees as a paste produced by mixing ground pollen pellets with distilled water to achieve a uniform consistency.

### Inoculation treatment

Experimental adult worker bumble bees were obtained from a single commercial *B. impatiens* colony that was determined to be uninfected by screening five workers using the methods described in *Preparing inoculation treatments*. Workers were removed from the colony and placed into individual plastic containers (7.5 cm × 10 cm × 5 cm) with mesh screen flooring. We starved the bees for 3–5 h and then fed each a 10 μL drop of either the *C. bombi* inoculum or sham control; bees were assigned at random to inoculation treatment. The dose of *C. bombi* inoculum contained 12,000 *C. bombi* cells, which is within the concentration range bees are exposed to when foraging on flowers in the wild [88]. Only bees that consumed the entire droplet (*n *= 120; 60 with *C. bombi* and 60 with sham inoculum) were used in the experiment.

All bees were then randomly assigned within inoculation treatment to either the sunflower (*n* = 60) or wildflower pollen diet (*n* = 60). Each day we fed bees fresh pollen paste of their assigned treatment, packed into an inverted lid of a 1.5 mL microcentrifuge tube and 1 mL of 30% sucrose via a filled and inverted plastic 1.5 mL microcentrifuge tube plugged with cotton (Richmond Dental & Medicine, Charlotte, NC, USA). We harvested tissue samples for RNA extraction 72 h post-inoculation. We chose 72 h post-inoculation because our results indicated that diet-driven differences in infection become statistically discernible between 72 and 96 h (Fig. 1B and 1C; see Supplementary Text: Timing of sunflower pollen effect methods). Moreover, a recent study demonstrated that consuming sunflower pollen for approximately the first 72 h or for 7 days after inoculation both reduced *C. bombi* intensity in bumble bees compared with control pollen [55]. We harvested tissue samples for RNA extraction from five workers per treatment that had the greatest average daily rate of pollen consumption (see *Pollen consumption* below). In total, we sequenced 20 samples: 5 replicates for each inoculation treatment and pollen diet. The remaining 100 bees (referred to as non-RNAseq-bees) were reserved to indirectly determine inoculation efficacy by measuring *C. bombi* infection.

### Pollen consumption

Previous work showed that consuming higher concentrations of sunflower pollen had a stronger medicinal effect [23]. Because it was not feasible to control how much pollen an individual bee consumed in our study, estimating pollen consumption was important to effectively model the relationship between diet and gene expression. To estimate consumption of pollen over the 72-h period, we recorded the weight of each pollen feeder each day before placing it into the container with the bee and also 24 h later. We accounted for feeder weight change caused by evaporation by placing an additional 30 pollen and nectar feeders (15 per pollen type) into containers that lacked a bee. Each day bees were provided fresh sucrose and pollen, yielding three days (post inoculation) of pollen consumption and evaporation measurements. We were not able to estimate nectar consumption because nectar feeders often leaked.

All statistical analyses using linear models were conducted with R version 4.0.2 [89]. To estimate pollen consumption, we calculated evaporation-adjusted net consumption based on change in weight of the pollen feeder for each bee per day. Using the evaporation controls, we fit separate linear regressions for each day and pollen type, with initial weight regressed against weight 24-h later. We then used the *predict* function in R to calculate an evaporation-adjusted feeder weight, yielding a net consumption estimate for each bee each day. Consumption variables (day 1, day 2, day 3, average daily rate (mg/day) and total) were strongly correlated based on Pearson's product moment correlations (t > 4.538, df = 34, *p* < 0.001 for all combinations). We thus focused solely on average daily pollen consumption rate for all gene expression analyses (see *Differential gene expression analysis*), as this was the metric used to select bees for RNA sequencing. We used ANOVA to test for differences in average daily pollen consumption rate between pollen diets and inoculation treatments for RNAseq bees. Model estimated means and Tukey-adjusted pairwise comparisons were obtained using the “emmeans” package [90].

### Efficacy of inoculation

To verify that bees inoculated with *C. bombi* were infected and that sunflower pollen reduced *C. bombi* infection relative to wildflower pollen, we measured *C. bombi* prevalence and infection intensity of a random subset of the remaining bees that were not selected for RNA extraction, but also consistently consumed their pollen treatments over the first 72 h [n(sunflower pollen) = 15 bees, n(wildflower pollen) = 18 bees]. Each bee was dissected and *C. bombi* cells were counted as in *Preparing inoculation treatments*, with the addition that all tools were washed with 70% ethanol and thoroughly dried between bees to prevent cross-contamination. We measured prevalence as the presence (1 or more *C. bombi* cells) or the absence of *C. bombi* cells per 0.02 μL sample, and *C. bombi* infection intensity as the number of flagellate *C. bombi* cells per 0.02 μL. We also removed the right forewing of each bee to measure marginal cell length, a proxy for bee size [91].

We used generalized linear models to analyze how pollen diets affected *C. bombi* infection prevalence and intensity. *Crithidia bombi* prevalence models were fit with a binomial distribution and infection intensity models were fit with a negative binomial distribution using the “MASS” package [92].

### RNA extractions and sequencing

For bees selected for RNA sequencing, at 72 h post-inoculation, the bees were anesthetized in a container of dry ice for 2 min. Using flame-sterilized forceps, we removed the abdomen of each anesthetized bee and placed it into a sterile 2 mL microcentrifuge tube with 2 mL of RNA-stabilizing reagent (RNAlater; ThermoFisher, Waltham, MA, USA; cat. No. AM7021). Each abdomen was slightly torn open with forceps for the RNA-stabilizing reagent to fully saturate the tissue sample and stored at 4 °C for 24 h. All samples were then kept in a -80 °C freezer until RNA extraction. *Crithidia bombi* is a gut pathogen in bumble bees, and since our interests were in the effects of diet, we focus the sequencing on abdominal tissues. We did not use whole-bees to avoid potential tissue-specific gene expression patterns (e.g., differences between brain and gut gene expression), which has been shown in other insects [93] and may make it difficult to disentangle gut-specific responses.

Total RNA samples (*n* = 20 samples: 5 replicates for each inoculation treatment and pollen diet) were submitted to the NCSU Genomic Sciences Laboratory for Illumina RNA library construction and sequencing. Purification of messenger RNA (mRNA) was performed using oligo-dT beads in the NEBNExt Poly(A) mRNA Magnetic Isolation Module. Complementary DNA (cDNA) libraries for Illumina sequencing were constructed using the NEBNext Ultra Directional RNA Library Prep Kit (NEB) and NEBNext Mulitplex Oligos for Illumina (NEB) using the manufacturer-specified protocol. Double-stranded cDNA was purified, end repaired, and “a-tailed” for adaptor ligation. Following ligation, samples were processed for a final fragment size (adapters included) of 400–550 bp using sequential AMPure XP bead isolation (Beckman Coulter, USA). Prior to library construction, RNA integrity, purity, and concentration was assessed using an Agilent 2100 Bioanalyzer with an RNA 6000 Nano Chip. Library enrichment was performed and specific indexes for each sample were added during the protocol-specified PCR amplification. The amplified library fragments were purified and checked for quality and final concentration using an Agilent 2100 Bioanalyzer with a High Sensitivity DNA chip. The final quantified libraries were pooled in equimolar amounts for clustering and sequencing on an Illumina NextSeq 500 DNA sequencer, using a 75 bp × 2 single end sequencing reagent kit. The software package Real Time Analysis was used to generate raw bcl (base call files), which were then de-multiplexed by sample into fastq files. Low-quality bases and adapter sequences were removed from raw sequence data for each sample using the Trimmomatic software package. Clean reads were mapped to the *B. impatiens* genome (BIMP 2.2) with HiSat2 version 2.1.0 [94] using default parameters. Gene expression was quantified using StringTie version 2.0 [95] to determine the number of reads uniquely mapping to exons and summed at the transcript level using gene features annotated in the NCBI *B. impatiens* annotation file (BIMP 2.2; GCA_000188095.4). Here after, for each transcript product mentioned throughout we report NCBI RefSeq protein accession IDs for *B. impatiens* or blast top hit taxa if *B. impatiens* was unavailable.

### Differential gene expression analysis

We used a negative binomial generalized linear model using DESeq2 version 1.28.1 [96] in R to test for differences in gene expression between treatments. We tested for effects of treatment (pairwise comparisons of pollen diet and inoculation treatment) on gene expression and included pollen consumption rate as a continuous covariate to control for variation caused by differences in average daily pollen consumption among bees. We used the Wald test to assess the significance of differentially expressed transcripts (DETs) and corrected for multiple testing using the Benjamini–Hochberg method with a cutoff of FDR < 0.05. Shrunken log2 fold changes for normalized transcript counts were obtained using lfcShrink function in DESeq2 with a shrinkage estimator based on a normal prior [96].

### Machine learning analysis

Transcriptomic data often suffers from the ‘curse of dimensionality’ due to having many more features than samples [97]. Small sample sizes and the rapid loss of degrees of freedom thus make it a poor fit for traditional linear statistics, like regression and ANOVA [98]. Standard analyses typically use multiple testing corrections to control false discovery rate (FDR). This method does not consider the highly interactive system of the transcriptome and often fails to detect small changes in gene expression [99]. Machine learning, or artificial intelligence tools, can be used to address these challenges by building models from the data rather than fitting the data to rigid models.

We applied support vector machines (SVM) for classification using Weka 3.8.4 [100] to the gene expression profiles of each pairwise treatment comparison. Classification in machine learning is the task of learning to distinguish data points that belong to two or more categories in a dataset. Feature selection techniques can then be used to select a reduced number of variables that can maintain accurate classification. For example, comparing infected bumble bees fed either sunflower or wildflower pollen, support vector machines can be used to determine how well pollen diet can be classified from gene expression profiles. Feature selection can then be used to determine a reduced set of transcripts that accurately classify pollen diet, and are thus important (i.e., akin to statistical significance). To optimize data dimensionality for feature selection, we first selected a subset of transcripts from each pairwise treatment from the DESeq2 models that were differentially expressed based on an uncorrected p-value < 0.05. Transcripts (attributes) were then ranked using the InfoGain attribute evaluator and Ranker search method. This process evaluates the worth of an attribute by measuring the information gain with respect to the treatment [101]. Specifically, InfoGain measures the difference in the Shannon's entropy of the system H(S) before a new attribute X is introduced, and H(S|X) is the entropy of the system after the attribute X has been introduced. We then created a series of ranked datasets, each including a subset of the top-ranked transcripts in a serial manner.

Preliminary classifier runs demonstrated that a support vector machine SMO, using the tenfold (stratified hold-out) cross-validation method, correctly classified an average of 82.90% (SD = 27.68%) of bee treatment effects based on gene expression profiles, and thus was used in our analyses. SMO implements John Platt’s sequential minimal optimization algorithm for training a support vector classifier by globally replacing all missing values and transforming nominal attributes, in our case transcripts, into binary attributes [102]. The machine-learning algorithm SMO has been successfully used to analyze gene expression profiles [103]. We used the ranked data sets to train the SMO algorithm using both the tenfold (stratified hold-out) and 66% split cross-validation method. For each data set, we repeated model training 100 times and used the classification performance metrics percent correct classification (%CC) and kappa statistic (k) to evaluate model performance.

We tested the efficacy of the optimized SMO model using a negative control method for machine learning. We first created 10 randomized data sets using the DESeq2 normalized counts with treatment randomly assigned. We then re-ran the SMO model training using the number of attributes that provided the best classification. Since there are always two class types, the predicted correct classification rate from random assignment should be approximately 50%, based on the Law of Probability, and the kappa statistic should be close to zero for a randomized negative control to demonstrate that true learning occurred in the optimized SMO models. This approach is detailed in previous studies [104, 105].

### Gene ontology enrichment analysis

Transcript descriptions and gene ontology (GO) annotations for transcript sequences were obtained using OmicsBox version 1.4.11 software (https://www.biobam.com/omicsbox/). First, a BED formatted file of transcript coordinates was parsed from the NCBI BIMP 2.2 Annotation release. We then used bedtools version 2.29.2 [106] to extract nucleotide sequences based on the BED file coordinates. A BLASTX search was then performed with an E-value of 10^–25^ against all arthropod sequences in the NCBI non-redundant database, with the number of hits restricted to 20, followed by GO mapping and annotation for the resulting hits. We then ran InterProScan annotation for the sequences using the default settings and merged InterProScan GO annotations with BLASTX annotations. GO enrichment analysis was performed for all treatment comparisons to find significantly (FDR < 0.05) enriched GO biological process and molecular function terms in the test set of DEGs with respect to the reference set. We used the publicly available databases GeneCards [107] and UniProtKB/Swiss-Prot [108] as additional primary sources of information about DEGs.

### IPA canonical pathway analysis

We used Qiagen Ingenuity Pathway Analysis (IPA) software to further interpret the differential expression of transcripts in each treatment pairwise comparison. IPA Knowledge Base maintains a large set of databases that consist of curated metabolic and signaling pathways. Transcripts were manually mapped to human, mouse, or rat ortholog gene IDs or those of other species based on UniProtKB accession numbers for use in IPA. We also performed an additional blastx for all transcripts using the same methods described in *Gene Ontology enrichment analysis*, but restricted to the *Homo sapiens*, *Mus* and *Rattus* taxonomies. Using these two different methods allowed us to double-check ambiguous orthologous gene symbols. We then performed a Core Analysis in IPA to determine enrichment of relevant canonical metabolic and signaling pathways based on gene expression patterns. We repeated Core Analysis for each pairwise treatment comparison based on the list of important transcripts identified using machine learning. The significance of the association between each gene set and a Canonical Pathway was determined from a p-value of overlap calculated using a right-tailed Fisher’s Exact Test. In addition, IPA calculates a z-score based on the gene expression fold change values of each gene to estimate the state of activation or inhibition of each pathway. We report gene symbols (*Homo sapiens*, *Mus* or *Rattus*) for each gene product mentioned hereafter in the IPA canonical pathway results.

## Supplementary Information

**Additional file 1.**

## Data Availability

The datasets generated and/or analyzed during the current study are available in the NCBI Sequence Read Archive repository (BioProject ID: PRJNA780223), [https://www.ncbi.nlm.nih.gov/bioproject/ PRJNA780223].

## Abbreviations

DET: Differentially expressed transcripts
FDR: False Discovery Rate
SD: Standard Deviation
SMO: Sequential minimal optimization
IPA: Ingenuity Pathway Analysis
SVM: Support Vector Machines
ABC: ATP-binding cassette
CYP: Cytochrome P450
LFC: Log Fold Change
AUROC: Area Under the Receiver Operating Characteristics
EAJS: Epithelial Adherens Junction Signaling pathway
