# Supplementary material for: Differential bumble bee gene expression associated with pathogen infection and pollen diet
Source: BMC Genomics. 2023 Mar 29;24:157. doi: 10.1186/s12864-023-09143-5 (PMC10053769; doi:10.1186/s12864-023-09143-5)
Supplement: Supplementary file 1 — Additional file 1. [file 12864_2023_9143_MOESM1_ESM.docx]

Supporting Information

Differential bumble bee gene expression associated with pathogen infection and pollen diet

Jonathan J. Giacomini^1*^, Lynn S. Adler^2a^, Benjamin J. Reading^1b^, and Rebecca E. Irwin^1c^

^1^ Department of Applied Ecology, North Carolina State University, Raleigh, NC, 27695 USA

^2^ Department of Biology, University of Massachusetts Amherst, Amherst, MA 01003 USA

*Corresponding author: Jonathan J. Giacomini, email: jjgiacom@ncsu.edu, ORCID: 0000-0002-0151-894X

^a^ ORCID: 0000-0003-2125-5582

^b^ ORCID: 0000-0002-0778-4069

^c^ ORCID: 0000-0002-1394-4946

**Supplementary Text: Timing of sunflower pollen effect methods.**

***Experimental Design.*** The purpose of this experiment was to help determine the timing of the effect of sunflower pollen on *Crithidia* *bombi* infection within *Bombus impatiens* workers. The results were used in conjunction with another study (1) to determine when the abdomens of each experimental bee should be dissected for RNA sequencing. Worker bumble bees were hand-inoculated with a standardized dose of live *Crithidia* cells mixed into a sucrose solution and provided either sunflower pollen or control wildflower pollen (hereafter referred to as pollen type) for 24 hours, 48 hours, 72 hours, 96 hours or 168 hours (hereafter referred to as timing treatment). Each timing treatment corresponds to an amount of time in which a bee was allowed to consume pollen. For the remainder of the time (until 168 hours post-inoculation), a wildflower pollen mixture was provided to each bee. This protocol ensured that all bees were dissected on the same day, which allowed *Crithidia* infection intensity to reach representative levels within all bees for comparison.

***Preparing pollen diets.*** We prepared two pollen diet treatments – sunflower and control wildflower. Honey bee-collected sunflower pollen pellets were obtained from Changge Hauding Wax Industry (China) and sorted by color to remove impurities. We verified a pure batch of sunflower pollen by staining five samples with basic fuschin dye (2) and visually confirming only sunflower pollen was present with a compound microscope at 400X magnification. Honey bee-collected mixed wildflower pollen pellets were obtained from Koppert Biological Systems (Howell, MI, USA) and microscopically confirmed to contain < 5% Asteraceae pollen, identified by having spines on the exine (3). Experimental pollen diets were provided to bees as a paste produced by mixing ground pollen pellets with distilled water to achieve a uniform consistency.

***Inoculation methods.*** Three *Bombus impatiens* colonies were purchased from Koppert Biological Systems (Howell, MI, USA). We screened each colony for pre-existing *Crithidia* infection two days after receipt by dissecting five workers from each colony using previously established protocols (4–6). Colonies were fed with 30% sucrose solution and mixed wildflower pollen throughout their lifetimes and housed in a dark room at 21 – 24ºC and ~50% rh. We removed 36 workers from two of the colonies and 60 workers from the third colony (total = 132 workers). Each bee was placed into a 7-dram snap cap vial and allowed to rest on the lab bench for approximately 4 hours. This resting period starves the bees and helps facilitate consumption of the inoculum. During the starving period, we made *Crithidia* inoculum using an established protocol (4,6,7). Briefly, bee digestive tracts of 15 workers, excluding the honey crop, were removed with forceps, placed into 1.5 mL microcentrifuge tubes with 300 μL of distilled water, and ground with a pestle. We allowed each sample to rest at room temperature for 4-5 hours so that gut material settled and the *Crithidia* cells could ascend into the supernatant. *Crithidia* cells were counted from a 0.02 μL sample of supernatant per bee with a Neubauer hemacytometer under a compound light microscope at 400X magnification. We then mixed 150 μL of the supernatant with distilled water to achieve a concentration of 2400 cells μL^-1^. The sample was then mixed with an equal volume of 50% sucrose solution to yield inoculum with 1200 cells μL^-1^ in 25% sucrose. After the starving period, each bee was fed a 10 μL drop of a 25% sucrose solution that contained 1200 cells per μL (Total cells = 12,000). Consumption of the inoculum was visually confirmed for each bee. Each bee was randomly assigned to a pollen type (sunflower or wildflower pollen) and a timing treatment, then housed in a 7.6 cm x 12.7 cm plastic container with a mesh bottom for the remainder of the experiment. All bees were fed fresh sucrose and their respective pollen daily until dissection 168 hours post-inoculation.

***Pollen Consumption*.** To ensure that all bees consumed pollen, the amount of daily pollen consumption (mg/day) was measured for each bee. On a day-by-day basis, bees that consumed pollen, as determined by a net positive amount of pollen consumption after accounting for evaporation, were randomly assigned to a time treatment (24 hours, 48 hours, 72 hours, or 96 hours) or allowed to continue on in the experiment until 168 hours post-inoculation. To estimate net pollen consumption, we produced a set of evaporation controls for each feeding day by including 10 bee containers alongside the experimental bees that contained 6 pollen feeders each (3 sunflower and 3 control wildflower) and a nectar feeder, but lacked a bee. We calculated evaporation-adjusted net consumption for each bee by fitting separate linear regressions for each day and pollen type, with initial weight of evaporation control pollen feeder regressed against the weight of the evaporation control pollen feeder 24-hr later. We then used the *predict* function in R to calculate an evaporation-adjusted feeder weight for feeders provided to each bee, yielding a net consumption estimate for each bee each day. Bees that did not consume pollen (net negative pollen consumption) at any point during their timing treatment were removed from the experiment. In other words, only bees that consumed a net positive amount of pollen for each day during their respective timing treatment were included in the final analysis. This method allowed us to infer effects of sunflower pollen consumption and avoid confounding effects of staggered or missing consumption throughout the treatment feeding period. We were unable to accurately estimate nectar consumption and thus excluded nectar consumption from the analysis.

***Measuring parasite load.*** Each bee was dissected as in *Inoculation methods*, with the addition that all tools were washed with 70% ethanol and thoroughly dried between bees to prevent cross-contamination. *Crithidia* cells from a 0.02 μL sample of supernatant per bee were counted with a Neubauer hemacytometer at 400X magnification with a compound light microscope (4,6,7). We measured prevalence as the presence (1 or more *Crithidia* cells) or the absence of *Crithidia* cells per 0.02 μL of each sample, and *Crithidia* infection intensity as the number of flagellate *Crithidia* cells per 0.02 μL. We also removed the right forewing of each bee to measure marginal cell length, a proxy for bee size (8).

***Statistical analyses.*** Statistical analyses were conducted using R version 4.0.2 (9). We used generalized linear models to analyze how pollen diets and timing treatments affected *Crithidia* infection prevalence and intensity. The *Crithidia* prevalence model was fit with a binomial distribution, and the infection intensity model was fit with a negative binomial distribution using the “MASS” package (10). Pollen type, timing treatment, and the interaction between pollen type and timing treatment were included as fixed effects in each model. Pairwise contrasts were evaluated separately for each combination of pollen type and timing treatment using the “emmeans” package (11). Due to low sample size (Table S4.11), we were unable to fit mixed-effect models with a random effect term for colony origin. In total, 45 sunflower-fed and 42 wildflower-fed bees were included in the analysis, which resulted in 5 to 13 bees per pollen type per timing treatment.

***Timing of sunflower pollen effect results.*** We detected *Crithidia* infection in approximately 85% of bees fed control wildflower-fed throughout the entire experiment (168 hours post-inoculation; Figure S1a), indicating successful inoculation. In comparison, only 21% of sunflower-fed bees had detectable infection by the end of the experiment. A generalized linear model indicated a significant effect of pollen type on *Crithidia* prevalence (χ^2^ = 14.101, df = 2, p = 0.001), but not the timing treatment or their interaction (χ^2^ < 7.2, p > 0.2; df = 5 and 4, respectively). Pairwise contrasts indicated a significant difference in prevalence between sunflower pollen and control wildflower pollen for the timing treatments of 96 hours and 168 hours post-inoculation (t = -2.417, p = 0.018; t = -2.874, p = 0.005; respectively), but not 24 hours, 48 hours or 72 hours post-inoculation (t < 0.001, p > 0.999; for all). Similar to prevalence, infection intensity was greatest in bees fed control wildflower-fed bees throughout the entire experiment (168 hours post-inoculation; Figure S1b). The generalized linear model indicated a significant effect of pollen type and timing treatment on infection intensity (χ^2^ > 30.549, p < 0.0001 for both; df = 1 and 4, respectively), but not their interaction (χ^2^ = 0.993, df = 4, p = 0.911). Similar to prevalence, pairwise contrasts indicated a significant difference in infection intensity between sunflower pollen and control wildflower pollen at 96 hours and 168 hours post-inoculation (t = -2.890, p = 0.004; t = -3.979, p = 0.0001; respectively), but not at 24 hours, 48 hours or 72 hours post-inoculation (t < 0.004, p > 0.997; for all). Taken together, these results suggest that *Crithidia* growth within host bumble bees diverges due to diet between 72 and 96 hours post-inoculation.

**Supplemental Figures and Tables**

**Figure S1**. Species distribution of top BLAST hits from a blastx (OmicsBox) search using E-value of 10^-25^ against all arthropod sequences in the NCBI non-redundant database, with the number of hits restricted to 20. Out of 17077 hits the greatest number of top BLAST hits were found in *B. impatiens*, with the top five being *Bombus*, thus giving us confidence in our RNA sequence read quality.

**Figure S2**. Machine learning model performance of support vector machine (SMO) ability to correctly classify pollen diet (sunflower (S) or wildflower (W)) based on transcriptomes of *Bombus impatiens* workers infected (I) with *Crithidia bombi*. Two cross-validation methods were used: 10-fold stratified hold-out (blue) and 66% percent split (red). Vertical dashed blue lines indicate convergence between both validation methods in the number of genes that produced greatest model performance. Genes were selected from the DESeq2 models that were differentially expressed based on an un-corrected p-value < 0.05 and ranked based on Shannon's entropy using the InfoGain attribute evaluator and Ranker search method.

**Figure S3**. GO terms (biological processes and molecular activity) enriched in the machine learning DEG list for infected bees fed either sunflower or wildflower pollen. All terms are significant at an FDR < 0.05.

**Figure S4**. Machine learning model performance of support vector machine (SMO) ability to correctly classify pollen diet (sunflower (S) or wildflower (W)) based on transcriptomes of control (C) un-infected *Bombus impatiens*. Two cross-validation methods were used: 10-fold stratified hold-out (blue) and 66% percent split (red). Vertical dashed blue lines indicate convergence between both validation methods in the number of genes that produced greatest model performance. Genes were selected from the DESeq2 models that were differentially expressed based on an un-corrected p-value < 0.05 and ranked based on Shannon's entropy using the InfoGain attribute evaluator and Ranker search method.

**Figure S5.** GO terms (biological processes and molecular activity) enriched in the machine learning DEG list for un-infected bees fed either sunflower or wildflower pollen. All terms are significant at an FDR < 0.05.

**Figure S6.** Machine learning model performance of support vector machine (SMO) ability to correctly classify infection treatment (inoculated with sham control (C) or *Crithidia bombi* (I)) based on transcriptomes of *Bombus impatiens* fed sunflower pollen (S). Two cross-validation methods were used: 10-fold stratified hold-out (blue) and 66% percent split (red). Genes were selected from the DESeq2 models that were differentially expressed based on an un-corrected p-value < 0.05 and ranked based on Shannon's entropy using the InfoGain attribute evaluator and Ranker search method.

**Figure S7.** Machine learning model performance of support vector machine (SMO) ability to correctly classify infection treatment (inoculated with sham control (C) or *Crithidia bombi* (I)) based on transcriptomes of *Bombus impatiens* fed wildflower pollen (W). Two cross-validation methods were used: 10-fold stratified hold-out (blue) and 66% percent split (red). Vertical dashed blue lines indicate convergence between both validation methods in the number of genes that produced greatest model performance. Genes were selected from the DESeq2 models that were differentially expressed based on an un-corrected p-value < 0.05 and ranked based on Shannon's entropy using the InfoGain attribute evaluator and Ranker search method.

**Table S1**. Transcriptome assembly statistics for *Bombus impatiens* workers inoculated with either a sham control (C) or *Crithidia bombi* (I) and fed either sunflower pollen (S) or wildflower pollen (W). Clean reads were mapped to the *B. impatiens* genome (NCBI *B. impatiens* BIMP 2.2; GCA_000188095.4) with HiSat2 version 2.1.0.

| Inf. | Pollen | Clean reads | Uniquely mapped reads | Uniquely mapped rate | Reads mapped >1 times | Mapped >1 time rate | Un-mapped reads | Un-mapped rate | Overall map rate |
| --- | --- | --- | --- | --- | --- | --- | --- | --- | --- |
| C | S | 23335668 | 21316818 | 91.35% | 275766 | 1.18% | 1743084 | 7.47% | 92.53% |
| C | S | 22280940 | 20208002 | 90.70% | 240041 | 1.08% | 1832897 | 8.23% | 91.77% |
| C | S | 20563334 | 17855936 | 86.83% | 232087 | 1.13% | 2475311 | 12.04% | 87.96% |
| C | S | 21876134 | 19955912 | 91.22% | 274382 | 1.25% | 1645840 | 7.52% | 92.48% |
| C | S | 50201346 | 46094685 | 91.82% | 569847 | 1.14% | 3536814 | 7.05% | 92.95% |
| I | S | 23857146 | 21456697 | 89.94% | 319383 | 1.34% | 2081066 | 8.72% | 91.28% |
| I | S | 21081260 | 19202098 | 91.09% | 255888 | 1.21% | 1623274 | 7.70% | 92.30% |
| I | S | 29145053 | 26418761 | 90.65% | 317152 | 1.09% | 2409140 | 8.27% | 91.73% |
| I | S | 60011808 | 54555874 | 90.91% | 697392 | 1.16% | 4758542 | 7.93% | 92.07% |
| I | S | 15836186 | 14128277 | 89.22% | 173275 | 1.09% | 1534634 | 9.69% | 90.31% |
| C | W | 16426934 | 14658243 | 89.23% | 158521 | 0.97% | 1610170 | 9.80% | 90.20% |
| C | W | 41982749 | 38203936 | 91.00% | 421431 | 1.00% | 3357382 | 8.00% | 92.00% |
| C | W | 19376962 | 17621534 | 90.94% | 234904 | 1.21% | 1520524 | 7.85% | 92.15% |
| C | W | 31979220 | 29143727 | 91.13% | 362991 | 1.14% | 2472502 | 7.73% | 92.27% |
| C | W | 19639797 | 17274582 | 87.96% | 241821 | 1.23% | 2123394 | 10.81% | 89.19% |
| I | W | 25383002 | 22830985 | 89.95% | 264720 | 1.04% | 2287297 | 9.01% | 90.99% |
| I | W | 20691247 | 18770572 | 90.72% | 253799 | 1.23% | 1666876 | 8.06% | 91.94% |
| I | W | 42245908 | 38187736 | 90.39% | 463582 | 1.10% | 3594590 | 8.51% | 91.49% |
| I | W | 13702311 | 12166254 | 88.79% | 150068 | 1.10% | 1385989 | 10.12% | 89.88% |
| I | W | 22198707 | 20172087 | 90.87% | 265959 | 1.20% | 1760661 | 7.93% | 92.07% |
| Mean | | 27090786 | 24511136 | 90.23% | 308650 | 1.14% | 2270999 | 8.62% | 91.38% |
| Std. Dev. | | 12255014 | 112651623 | 1.25% | 137170 | 0.09% | 891882 | 1.27% | 1.27% |

**Table S2.** Differentially expressed genes between infected sunflower- and infected wildflower-fed bees based on the DESeq2 model (FDR < 0.05). Gene descriptions based on top BLAST hits (E-value of 10^-25^) against all arthropod sequences in the NCBI non-redundant database.

| StringTie ID | Fold Change (log2) | FDR | Description |
| --- | --- | --- | --- |
| MSTRG.21535 | 4.8157 | 0.0270 | hymenoptaecin |
| MSTRG.20285 | -2.4744 | 0.0355 | prohormone-3 |
| MSTRG.11358 | 21.2532 | 0.0000 | glutamate receptor 3.2-like |
| MSTRG.8149 | 30.1294 | 0.0000 | uncharacterized protein LOC117204693 |
| MSTRG.3654 | -2.0130 | 0.0153 | PREDICTED: uncharacterized protein LOC105662916 |
| MSTRG.14101 | 5.9837 | 0.0380 | uncharacterized protein LOC117233211 isoform X8 |
| MSTRG.8725 | -1.4158 | 0.0380 | uncharacterized protein LOC100748164 isoform X3 |
| MSTRG.9723 | 3.9849 | 0.0380 | uncharacterized protein LOC100749322 isoform X2 |
| MSTRG.3174 | 6.5770 | 0.0153 | cytochrome P450 9e2-like |
| MSTRG.11066 | 11.4536 | 0.0017 | probable endochitinase |
| MSTRG.5278 | -1.8675 | 0.0425 | TWiK family of potassium channels protein 7 |
| MSTRG.14467 | 44.3295 | 0.0000 | RNaN-directed DNaN polymerase from mobile element jockey-like |
| MSTRG.7865 | -36.7045 | 0.0000 | ejaculatory bulb-specific protein 3-like |
| MSTRG.19478 | 25.1746 | 0.0000 | glucose dehydrogeNaNse [FAD, quinone]-like |
| MSTRG.11147 | 8.0354 | 0.0000 | alkaline phosphatase 4-like |
| MSTRG.12016 | 4.7762 | 0.0110 | maltase A2 isoform X1 |
| MSTRG.6331 | -1.5704 | 0.0189 | enzymatic polyprotein endonuclease reverse |
| MSTRG.16362 | 1.2463 | 0.0355 | putative inorganic phosphate cotransporter |
| MSTRG.19383 | 23.0473 | 0.0078 | digestive cysteine proteinase 1 |
| MSTRG.872 | -2.8824 | 0.0001 | piggyBac transposable element-derived protein 4-like |
| LOC105680309 | 24.8898 | 0.0023 | probable ATP-dependent RNA helicase DDX46 |
| MSTRG.8718 | 6.6666 | 0.0056 | TM2 domain-containing protein |
| MSTRG.18222 | 0.8339 | 0.0149 | nicastrin isoform X2 |
| MSTRG.20614 | 5.8225 | 0.0149 | mitochondrial potassium channel ATP-binding subunit |
| MSTRG.14747 | 9.4394 | 0.0153 | beta-1,4-glucuronyltransferase 1 |
| MSTRG.4550 | -1.0866 | 0.0190 | ATP-binding cassette sub-family G member 1 |
| MSTRG.8519 | 7.3143 | 0.0223 | oxidation resistance protein 1 isoform X9 |
| MSTRG.8539 | 6.8131 | 0.0355 | NPC intracellular cholesterol transporter 2-like |
| MSTRG.8520 | 5.1741 | 0.0452 | oxidation resistance protein 1 isoform X6 |
| MSTRG.18428 | 0.6567 | 0.0467 | cholinephosphotransferase 1 isoform X3 |
| MSTRG.9363 | -1.4573 | 0.0467 | carbohydrate sulfotransferase 11 |
| MSTRG.362 | 46.4711 | 0.0000 | NaN |
| MSTRG.12884 | 29.4999 | 0.0000 | NaN |
| MSTRG.996 | 15.2676 | 0.0002 | NaN |
| MSTRG.2541 | -3.2737 | 0.0153 | NaN |
| MSTRG.3872 | 5.8753 | 0.0153 | serine protease inhibitor dipetalogastin |
| MSTRG.21434 | 5.1708 | 0.0212 | NaN |
| MSTRG.16497 | 21.2627 | 0.0256 | hypothetical protein ALC62_09346 |
| MSTRG.20557 | 4.8864 | 0.0380 | NaN |
| MSTRG.7729 | -1.2714 | 0.0425 | NaN |

**NaN = no available annotation.**

**Table S3.** Top-ranked 160 genes that differentiate infected sunflower- and infected wildflower-fed bees based on a 100% correct classification rate using Machine Learning. A subset of genes from the DESeq2 model with an uncorrected p-value < 0.05 were ranked (Rank) based on information gain with respect to the treatment using the InfoGain attribute evaluator and Ranker search method in Weka. Gene descriptions based on top BLAST hits (E-value of 10^-25^) against all arthropod sequences in the NCBI non-redundant database.

| StringTie ID | Fold Change (log2) | FDR | Rank | Description |
| --- | --- | --- | --- | --- |
| MSTRG.8520 | 5.1741 | 0.0452 | 1 | oxidation resistance protein 1 isoform X6 |
| MSTRG.11543 | 2.2353 | 0.3857 | 2 | cyclin-A2 |
| MSTRG.5752 | -1.1313 | 0.3471 | 3 | ABC transporter G family member 23 |
| MSTRG.13004 | -1.2471 | 0.2864 | 4 | flexible cuticle protein 12-like |
| MSTRG.14101 | 5.9837 | 0.0380 | 5 | uncharacterized protein LOC117233211 isoform X8 |
| LOC105681892 | -1.4653 | 0.3503 | 6 | uncharacterized protein LOC117233964 isoform X1 |
| MSTRG.11112 | -2.6979 | 0.1358 | 7 | endocuticle structural glycoprotein SgAbd-4-like |
| MSTRG.9141 | 1.8054 | 0.3051 | 8 | solute carrier organic anion transporter family member 1A5 isoform X3 |
| MSTRG.5223 | -2.3864 | 0.3477 | 9 | NaN |
| MSTRG.8539 | 6.8131 | 0.0355 | 10 | NPC intracellular cholesterol transporter 2-like |
| MSTRG.1888 | 2.9456 | 0.3657 | 11 | thioredoxin reductase 1, mitochondrial isoform X3 |
| MSTRG.15603 | -1.8601 | 0.1575 | 12 | probable serine/threonine-protein kinase samkC isoform X3 |
| MSTRG.2194 | 3.7519 | 0.2785 | 13 | NaN |
| MSTRG.10445 | 1.6140 | 0.3948 | 14 | Neuropilin and tolloid-like protein 1 |
| MSTRG.20639 | 4.9580 | 0.2468 | 15 | transmembrane protease serine 9-like |
| MSTRG.15194 | 2.8636 | 0.1990 | 16 | putative inhibitor of apoptosis isoform X2 |
| MSTRG.5323 | -1.0620 | 0.1990 | 17 | Nose resistant to fluoxetine protein 6 |
| MSTRG.7877 | 2.1562 | 0.2441 | 18 | peptide transporter family 1 isoform X2 |
| MSTRG.3173 | 2.3805 | 0.0763 | 19 | cytochrome P450 9e2-like |
| MSTRG.1167 | 4.0506 | 0.2864 | 20 | homeobox protein ARX-like |
| MSTRG.20082 | 2.3136 | 0.2740 | 21 | protein regulator of cytokinesis 1-like |
| MSTRG.11252 | 2.7142 | 0.2984 | 22 | protein spaetzle 4 |
| MSTRG.5775 | 1.7092 | 0.3560 | 23 | max dimerization protein 1 isoform X2 |
| MSTRG.6180 | 5.1841 | 0.1805 | 24 | aminopeptidase N |
| MSTRG.10178 | 4.3293 | 0.2409 | 25 | farnesol dehydrogenase-like |
| MSTRG.328 | 0.9206 | 0.4595 | 26 | cytochrome P450 9e2-like |
| MSTRG.10176 | 3.2853 | 0.1679 | 27 | farnesol dehydrogenase-like |
| MSTRG.11845 | 2.4372 | 0.4031 | 28 | probable multidrug resistance-associated protein lethal(2)03659 |
| MSTRG.7691 | 2.0235 | 0.4729 | 29 | NaN |
| MSTRG.3174 | 6.5770 | 0.0153 | 30 | cytochrome P450 9e2-like |
| MSTRG.10693 | 3.1739 | 0.2740 | 31 | Coiled-coil domain-containing protein 13 |
| MSTRG.5531 | -1.8260 | 0.4651 | 32 | uncharacterized protein LOC105189360 isoform X1 |
| MSTRG.128 | 1.6859 | 0.4363 | 33 | NaN |
| MSTRG.7209 | 2.3821 | 0.2639 | 34 | spectrin beta chain, non-erythrocytic 2 isoform X4 |
| MSTRG.20055 | 4.6991 | 0.2162 | 35 | Alpha-amylase 4N |
| MSTRG.4011 | 1.8716 | 0.4755 | 36 | sporozoite surface protein 2-like |
| MSTRG.3529 | -2.0199 | 0.4250 | 37 | homeobox protein rough |
| MSTRG.18721 | 2.7970 | 0.4063 | 38 | Haloacid dehalogenase-like hydrolase domain-containing protein 2 |
| MSTRG.16555 | 2.3924 | 0.4287 | 39 | NaN |
| MSTRG.20058 | 5.3531 | 0.2616 | 40 | Alpha-amylase-related protein |
| MSTRG.15815 | 1.3312 | 0.4646 | 41 | E3 ubiquitin-protein ligase MARCH5 isoform X1 |
| MSTRG.1625 | 4.3249 | 0.2785 | 42 | chymotrypsin-1 |
| MSTRG.18320 | 4.3598 | 0.3620 | 43 | maltase 2-like |
| MSTRG.8221 | 2.6658 | 0.0817 | 44 | probable ATP-dependent RNA helicase DDX28 |
| MSTRG.10703 | 4.9882 | 0.2409 | 45 | facilitated trehalose transporter Tret1-like |
| MSTRG.13224 | 1.8790 | 0.4421 | 46 | aconitate hydratase, mitochondrial |
| MSTRG.9658 | 1.9252 | 0.3182 | 47 | E3 ubiquitin-protein ligase MIB2 |
| MSTRG.13228 | 2.0740 | 0.2312 | 48 | piggyBac transposable element-derived protein 4-like |
| MSTRG.13229 | 3.0219 | 0.2740 | 49 | NaN |
| MSTRG.14832 | -1.2801 | 0.2035 | 50 | NaN |
| MSTRG.17560 | 3.0003 | 0.1791 | 51 | NaN |
| MSTRG.2166 | 1.5898 | 0.4171 | 52 | uncharacterized protein LOC100740206 |
| LOC100742005 | -3.0339 | 0.1232 | 53 | cytoplasmic dynein 2 heavy chain 1 |
| MSTRG.5369 | -1.8027 | 0.3725 | 54 | NaN |
| SWRh | 5.4940 | 0.4250 | 55 | opsin, ultraviolet-sensitive |
| MSTRG.8521 | 5.9837 | 0.1550 | 56 | oxidation resistance protein 1 isoform X7 |
| MSTRG.9087 | 2.8125 | 0.1861 | 57 | uncharacterized protein LOC100740339 isoform X3 |
| MSTRG.19203 | 1.2584 | 0.2188 | 58 | transmembrane protein KIAA1109 isoform X4 |
| MSTRG.7899 | 2.1301 | 0.3739 | 59 | actin-5C |
| MSTRG.17374 | 4.0633 | 0.2785 | 60 | zinc carboxypeptidase-like |
| MSTRG.16597 | 1.6762 | 0.3899 | 61 | nucleolysin TIAR |
| MSTRG.12194 | 5.5899 | 0.0699 | 62 | uncharacterized threonine-rich GPI-anchored glycoprotein PJ4664.02-like |
| MSTRG.2280 | -3.3144 | 0.1371 | 63 | hypothetical protein WH47_09040 |
| LOC105681602 | 2.4282 | 0.2434 | 64 | balbiani ring protein 3-like |
| MSTRG.1889 | 1.9904 | 0.4500 | 65 | thioredoxin reductase 1, mitochondrial isoform X2 |
| MSTRG.20788 | 1.5486 | 0.3310 | 66 | uncharacterized protein LOC117161787 |
| MSTRG.14270 | 0.7698 | 0.4488 | 67 | ADP-ribosylation factor 1 |
| MSTRG.13139 | 2.7488 | 0.1812 | 68 | 39S ribosomal protein S30, mitochondrial |
| MSTRG.1623 | 4.1955 | 0.2441 | 69 | NaN |
| MSTRG.3881 | 3.4611 | 0.4590 | 70 | Alpha-mannosidase 2 |
| MSTRG.5154 | 1.5403 | 0.4031 | 71 | ATP synthase subunit alpha, mitochondrial |
| MSTRG.5150 | 3.2674 | 0.2125 | 72 | ubiquitin carboxyl-terminal hydrolase 14 |
| MSTRG.11968 | 4.2689 | 0.3222 | 73 | maltase 1 |
| MSTRG.10179 | 2.8962 | 0.2149 | 74 | farnesol dehydrogenase-like |
| MSTRG.4519 | 4.0014 | 0.0852 | 75 | protein FAM151B isoform X2 |
| MSTRG.11969 | 4.5654 | 0.2740 | 76 | maltase 1 |
| MSTRG.2068 | 3.4386 | 0.4076 | 77 | partitioning defective 3 homolog isoform X3 |
| MSTRG.11965 | 6.5062 | 0.1150 | 78 | alpha-glucosidase-like isoform X1 |
| MSTRG.15256 | 1.3959 | 0.3208 | 79 | NaN |
| MSTRG.4704 | 3.9405 | 0.1679 | 80 | Zinc finger CCHC domain-containing protein 9 |
| MSTRG.8718 | 6.6666 | 0.0056 | 81 | TM2 domain-containing protein |
| MSTRG.825 | 1.8657 | 0.3894 | 82 | Catenin alpha |
| MSTRG.11065 | 2.8331 | 0.0699 | 83 | probable WRKY transcription factor protein 1 |
| MSTRG.15789 | 2.7328 | 0.2526 | 84 | protein angel |
| MSTRG.8955 | 3.4081 | 0.4514 | 85 | SET and MYND domain-containing protein 4-like |
| MSTRG.8369 | 2.4431 | 0.2468 | 86 | Proteasome subunit alpha type-4 |
| MSTRG.10733 | 2.4446 | 0.4262 | 87 | hexosaminidase D-like |
| MSTRG.8362 | 3.0582 | 0.3822 | 88 | tRNA modification GTPase GTPBP3, mitochondrial |
| MSTRG.4863 | -1.4910 | 0.2188 | 89 | Myosin light chain alkali |
| MSTRG.17373 | 4.8870 | 0.1358 | 90 | zinc carboxypeptidase-like |
| MSTRG.10731 | -1.0858 | 0.3083 | 91 | F-box/LRR-repeat protein 2 isoform X1 |
| MSTRG.19715 | 3.0427 | 0.3003 | 92 | farnesol dehydrogenase-like |
| MSTRG.4264 | -2.3357 | 0.3619 | 93 | hypothetical protein WN51_00414 |
| MSTRG.21292 | 3.7904 | 0.1813 | 94 | UDP-glucuronosyltransferase 2B17-like |
| MSTRG.3872 | 5.8753 | 0.0153 | 95 | serine protease inhibitor dipetalogastin |
| MSTRG.12016 | 4.7762 | 0.0110 | 96 | maltase A2 isoform X1 |
| MSTRG.14663 | -3.9664 | 0.4147 | 97 | Polypyrimidine tract-binding protein 2 |
| MSTRG.8913 | 2.9344 | 0.1659 | 98 | RRP12-like protein |
| MSTRG.2534 | 1.5899 | 0.3476 | 99 | RNA-directed DNA polymerase from mobile element jockey |
| MSTRG.9637 | 3.2075 | 0.1812 | 100 | lysosomal aspartic protease |
| MSTRG.17231 | 1.0199 | 0.2713 | 101 | uridine diphosphate glucose pyrophosphatase |
| MSTRG.19349 | 2.6199 | 0.3857 | 102 | Dopamine N-acetyltransferase |
| MSTRG.413 | 3.6691 | 0.2795 | 103 | adenylate cyclase type 2 isoform X2 |
| MSTRG.14772 | 4.7662 | 0.3075 | 104 | NaN |
| MSTRG.20638 | 8.2439 | 0.0763 | 105 | transmembrane protease serine 9-like |
| MSTRG.4575 | 3.2980 | 0.1288 | 106 | NaN |
| MSTRG.16410 | 1.2762 | 0.4180 | 107 | uncharacterized protein LOC100747659 isoform X4 |
| MSTRG.75 | -1.5967 | 0.4352 | 108 | insulin-like peptide receptor isoform X1 |
| MSTRG.14907 | 2.4632 | 0.1679 | 109 | NaN |
| MSTRG.16918 | 1.7859 | 0.4312 | 110 | E3 ubiquitin-protein ligase AMFR-like |
| MSTRG.17464 | 1.0745 | 0.4590 | 111 | uncharacterized protein LOC117162246 |
| MSTRG.13552 | 4.6985 | 0.3332 | 112 | protein mesh isoform X1 |
| MSTRG.5826 | 1.6990 | 0.3136 | 113 | NaN |
| MSTRG.9723 | 3.9849 | 0.0380 | 114 | uncharacterized protein LOC100749322 isoform X2 |
| MSTRG.13589 | 1.7596 | 0.2856 | 115 | NaN |
| MSTRG.7600 | -2.8205 | 0.4100 | 116 | NaN |
| MSTRG.14601 | 1.9655 | 0.2911 | 117 | NaN |
| MSTRG.13252 | 5.0504 | 0.2800 | 118 | carboxypeptidase B-like |
| MSTRG.502 | 1.2860 | 0.4500 | 119 | tyrosine-protein phosphatase non-receptor type 2 isoform X2 |
| MSTRG.3516 | 2.6846 | 0.4408 | 120 | NaN |
| MSTRG.9055 | -2.0090 | 0.4100 | 121 | NaN |
| MSTRG.1626 | 5.5920 | 0.0852 | 122 | chymotrypsin-1 |
| LOC105681186 | 2.8943 | 0.2785 | 123 | G-protein coupled receptor Mth2-like |
| MSTRG.8482 | 4.7322 | 0.3452 | 124 | chitinase-3-like protein 1 |
| MSTRG.890 | 4.8198 | 0.3112 | 125 | trypsin-1 isoform X1 |
| MSTRG.1577 | 2.0329 | 0.2468 | 126 | uncharacterized protein LOC117241145 isoform X2 |
| MSTRG.8481 | 6.3376 | 0.3657 | 127 | chitinase-3-like protein 1 |
| MSTRG.3866 | 2.2856 | 0.3012 | 128 | dnaJ homolog subfamily C member 11 |
| MSTRG.16385 | 2.0737 | 0.4683 | 129 | NaN |
| MSTRG.11414 | 4.8044 | 0.4702 | 130 | NaN |
| MSTRG.20549 | 3.5421 | 0.2820 | 131 | NaN |
| MSTRG.5529 | 1.6367 | 0.3343 | 132 | uncharacterized protein LOC102671994 |
| MSTRG.19259 | -1.2239 | 0.2964 | 133 | Retrovirus-related Pol polyprotein from transposon 17.6 |
| MSTRG.8211 | 1.9139 | 0.3081 | 134 | kxDL motif-containing protein CG10681 |
| MSTRG.12622 | 1.6176 | 0.4646 | 135 | NaN |
| MSTRG.695 | -3.7479 | 0.3051 | 136 | NaN |
| MSTRG.9636 | 3.3960 | 0.3332 | 137 | lysosomal aspartic protease |
| MSTRG.10504 | 1.9854 | 0.3551 | 138 | NaN |
| MSTRG.9634 | 4.2748 | 0.0577 | 139 | NaN |
| MSTRG.14366 | 1.2306 | 0.4514 | 140 | FACT complex subunit spt16 isoform X2 |
| MSTRG.9870 | 3.8966 | 0.2280 | 141 | cGMP-dependent protein kinase 1-like |
| MSTRG.6877 | 1.1398 | 0.2666 | 142 | cholinesterase isoform X1 |
| MSTRG.1099 | 4.3402 | 0.1581 | 143 | NaN |
| MSTRG.14600 | 1.0146 | 0.3973 | 144 | NaN |
| MSTRG.102 | 2.1415 | 0.3042 | 145 | V-type proton ATPase subunit D |
| MSTRG.18165 | 2.2897 | 0.3267 | 146 | uncharacterized protein LOC117152339 |
| MSTRG.20647 | -2.2205 | 0.4423 | 147 | NaN |
| MSTRG.424 | -1.6504 | 0.4512 | 148 | probable cytochrome P450 305a1 |
| MSTRG.1730 | 1.9570 | 0.4259 | 149 | NaN |
| MSTRG.9255 | 2.1475 | 0.2964 | 150 | carbonyl reductase [NADPH] 1-like |
| MSTRG.19626 | 5.0703 | 0.1328 | 151 | facilitated trehalose transporter Tret1 isoform X2 |
| MSTRG.18876 | 3.2078 | 0.4399 | 152 | meiosis regulator and mRNA stability factor 1 isoform X7 |
| MSTRG.1938 | -2.6216 | 0.0862 | 153 | circadian clock-controlled protein |
| MSTRG.4646 | 1.7799 | 0.3437 | 154 | NaN |
| MSTRG.889 | 4.6928 | 0.2468 | 155 | trypsin-3 |
| MSTRG.15486 | 1.7693 | 0.3940 | 156 | alanine--glyoxylate aminotransferase 2-like |
| MSTRG.9453 | 1.8858 | 0.4648 | 157 | complement component 1 Q subcomponent-binding protein, mitochondrial |
| MSTRG.20877 | -2.5394 | 0.2358 | 158 | mitotic apparatus protein p62 |
| MSTRG.5302 | 2.5046 | 0.3442 | 159 | 40S ribosomal protein S17 |
| MSTRG.4742 | -2.0975 | 0.4040 | 160 | sodium- and chloride-dependent GABA transporter 2-like |

**NaN = no available annotation.**

**Table S4**. Enrichment of canonical metabolic and signaling pathways identified by Qiagen’s IPA for pairwise treatment comparisons using the machine learning DEG lists. *Bombus impatiens* workers were inoculated with either a sham control (Uninfected) or *Crithidia bombi* (Infected) and fed either sunflower or wildflower pollen. Numeric values indicate log(p-value) for each gene set and a canonical pathway and red indicates significant enrichment based on a p-value of overlap calculated using a right-tailed Fisher’s Exact Test.

| **Canonical Pathways** | **Wildflower**: Infected vs. Uninfected | **Infected**: Sunflower vs. Wildflower | **Uninfected**: Sunflower vs. Wildflower |
| --- | --- | --- | --- |
| NRF2-mediated Oxidative Stress Response | 1.297 | 6.668 | 1.638 |
| SPINK1 Pancreatic Cancer Pathway | 0.000 | 5.571 | 0.000 |
| LPS/IL-1 Mediated Inhibition of RXR Function | 1.235 | 2.599 | 1.547 |
| Estrogen Biosynthesis | 0.000 | 4.311 | 0.880 |
| Epithelial Adherens Junction Signaling | 0.000 | 2.550 | 2.092 |
| Acetone Degradation I (to Methylglyoxal) | 0.000 | 3.410 | 1.021 |
| Aryl Hydrocarbon Receptor Signaling | 0.652 | 2.502 | 1.173 |
| Cellular Effects of Sildenafil (Viagra) | 0.000 | 2.531 | 1.187 |
| Neuroprotective Role of THOP1 in Alzheimer's Disease | 0.763 | 2.954 | 0.000 |
| Protein Ubiquitination Pathway | 0.459 | 2.446 | 0.795 |
| Nicotine Degradation III | 0.000 | 2.799 | 0.825 |
| Stearate Biosynthesis I (Animals) | 1.013 | 0.663 | 1.891 |
| Bupropion Degradation | 0.000 | 2.362 | 1.174 |
| Melatonin Degradation I | 0.000 | 2.633 | 0.772 |
| RhoA Signaling | 0.000 | 1.957 | 1.381 |
| Thioredoxin Pathway | 0.000 | 3.332 | 0.000 |
| Nicotine Degradation II | 0.000 | 2.566 | 0.751 |
| Vitamin-C Transport | 0.000 | 2.165 | 1.078 |
| Superpathway of Melatonin Degradation | 0.000 | 2.400 | 0.698 |
| Dilated Cardiomyopathy Signaling Pathway | 0.000 | 1.770 | 1.256 |
| Unfolded protein response | 0.892 | 1.369 | 0.685 |
| Tight Junction Signaling | 0.000 | 1.562 | 1.116 |
| Regulation of Actin-based Motility by Rho | 0.000 | 1.160 | 1.433 |
| LXR/RXR Activation | 0.745 | 0.422 | 1.357 |
| Hepatic Fibrosis / Hepatic Stellate Cell Activation | 0.000 | 1.445 | 1.037 |
| Polyamine Regulation in Colon Cancer | 0.000 | 1.653 | 0.825 |
| Sertoli Cell-Sertoli Cell Junction Signaling | 0.000 | 2.105 | 0.373 |
| Estrogen-Dependent Breast Cancer Signaling | 0.000 | 2.457 | 0.000 |
| Glycogen Degradation III | 0.000 | 1.152 | 1.300 |
| Remodeling of Epithelial Adherens Junctions | 0.000 | 1.591 | 0.795 |
| Protein Kinase A Signaling | 0.000 | 0.716 | 1.663 |
| Gap Junction Signaling | 0.000 | 1.376 | 0.991 |
| Mitochondrial Dysfunction | 0.000 | 2.309 | 0.000 |
| eNOS Signaling | 0.636 | 1.600 | 0.000 |
| Actin Cytoskeleton Signaling | 0.000 | 0.638 | 1.582 |
| Phagosome Maturation | 0.000 | 1.640 | 0.459 |
| Apelin Cardiomyocyte Signaling Pathway | 0.000 | 0.500 | 1.533 |
| Hepatic Cholestasis | 0.572 | 1.412 | 0.000 |
| Choline Degradation I | 1.974 | 0.000 | 0.000 |
| Huntington's Disease Signaling | 0.000 | 1.678 | 0.281 |
| Calcium Signaling | 0.000 | 0.252 | 1.701 |
| PRPP Biosynthesis I | 1.917 | 0.000 | 0.000 |
| Sorbitol Degradation I | 0.000 | 0.000 | 1.894 |
| PAK Signaling | 0.000 | 0.449 | 1.419 |
| Gα12/13 Signaling | 0.000 | 0.411 | 1.333 |
| Neuroinflammation Signaling Pathway | 0.000 | 1.485 | 0.241 |
| MSP-RON Signaling Pathway | 0.000 | 1.706 | 0.000 |
| Glycine Biosynthesis III | 0.000 | 1.664 | 0.000 |
| Thyroid Hormone Biosynthesis | 0.000 | 1.598 | 0.000 |
| Tetrahydrofolate Salvage from 5,10-methenyltetrahydrofolate | 0.000 | 0.000 | 1.596 |
| Superoxide Radicals Degradation | 0.000 | 0.000 | 1.596 |
| SPINK1 General Cancer Pathway | 0.000 | 1.580 | 0.000 |
| NAD Phosphorylation and Dephosphorylation | 1.523 | 0.000 | 0.000 |
| Assembly of RNA Polymerase III Complex | 0.000 | 0.000 | 1.484 |
| Urate Biosynthesis/Inosine 5'-phosphate Degradation | 1.463 | 0.000 | 0.000 |
| Guanosine Nucleotides Degradation III | 1.463 | 0.000 | 0.000 |
| Tyrosine Degradation I | 0.000 | 0.000 | 1.452 |
| DNA Double-Strand Break Repair by Homologous Recombination | 0.000 | 0.000 | 1.452 |
| Tryptophan Degradation to 2-amino-3-carboxymuconate Semialdehyde | 0.000 | 0.000 | 1.395 |
| Adenosine Nucleotides Degradation II | 1.394 | 0.000 | 0.000 |
| Crosstalk between Dendritic Cells and Natural Killer Cells | 0.000 | 1.351 | 0.000 |
| Mismatch Repair in Eukaryotes | 0.000 | 0.000 | 1.345 |
| Glycogen Degradation II | 0.000 | 0.000 | 1.345 |
| 5-aminoimidazole Ribonucleotide Biosynthesis I | 0.000 | 0.000 | 1.345 |
| Death Receptor Signaling | 0.000 | 1.318 | 0.000 |
| FXR/RXR Activation | 0.000 | 0.000 | 1.316 |
| Fatty Acid Activation | 0.000 | 0.000 | 1.300 |

**Table S5**. Differentially expressed genes between uninfected sunflower- and uninfected wildflower-fed bees based on the DESeq2 model (FDR < 0.05). Gene descriptions based on top BLAST hits (E-value of 10^-25^) against all arthropod sequences in the NCBI non-redundant database.

| String Tie ID | Fold Change (log2) | FDR | Description |
| --- | --- | --- | --- |
| MSTRG.19477 | 29.9440 | 0.0000 | glucose dehydrogenase [FAD, quinone]-like |
| MSTRG.9983 | 29.9894 | 0.0000 | uncharacterized protein LOC112212842 |
| MSTRG.14467 | 29.5606 | 0.0000 | RNA-directed DNA polymerase from mobile element jockey-like |
| MSTRG.20466 | -22.4594 | 0.0006 | NaN |
| MSTRG.20478 | -22.2347 | 0.0008 | dynein beta chain, ciliary-like |
| MSTRG.10459 | 18.5702 | 0.0018 | NaN |
| MSTRG.362 | -20.8330 | 0.0030 | NaN |
| MSTRG.17182 | 20.8841 | 0.0035 | putative odorant receptor 92a |
| MSTRG.15434 | 14.9761 | 0.0055 | NaN |
| MSTRG.19495 | 13.5107 | 0.0265 | jerky protein homolog-like |

**NaN = no available annotation.**

**Table S6.** Top-ranked 141 genes that differentiate uninfected sunflower- and uninfected wildflower-fed bees based on a 100% correct classification rate using Machine Learning. A subset of genes from the DESeq2 model with an un-corrected p-value < 0.05 were ranked (Rank) based on information gain with respect to the treatment using the InfoGain attribute evaluator and Ranker search method in Weka. Gene descriptions based on top BLAST hits (E-value of 10^-25^) against all arthropod sequences in the NCBI non-redundant database.

| String Tie ID | Fold Change (log2) | FDR | Rank | Description |
| --- | --- | --- | --- | --- |
| MSTRG.235 | -1.1252 | 1.0000 | 1 | lamin Dm0-like isoform X1 |
| MSTRG.7221 | -1.3343 | 1.0000 | 2 | NaN |
| MSTRG.9187 | 3.2432 | 1.0000 | 3 | NaN |
| MSTRG.502 | -1.2114 | 1.0000 | 4 | tyrosine-protein phosphatase non-receptor type 2 isoform X2 |
| MSTRG.4864 | -2.1114 | 1.0000 | 5 | myosin light chain alkali isoform X2 |
| MSTRG.17055 | 0.7114 | 1.0000 | 6 | NaN |
| MSTRG.15488 | -2.7218 | 1.0000 | 7 | vitellogenin |
| MSTRG.15843 | 1.9997 | 1.0000 | 8 | Transposon Tf2-9 polyprotein |
| MSTRG.14903 | -4.3002 | 1.0000 | 9 | distal membrane-arm assembly complex protein 2 |
| MSTRG.9958 | -1.0144 | 1.0000 | 10 | trifunctional purine biosynthetic protein adenosine-3 isoform X1 |
| MSTRG.21068 | 1.4029 | 1.0000 | 11 | uncharacterized protein LOC105666051 |
| MSTRG.16550 | -1.5799 | 1.0000 | 12 | Nucleolar and coiled-body phosphoprotein 1 |
| MSTRG.19724 | -1.9075 | 1.0000 | 13 | myosin regulatory light chain 2 |
| MSTRG.1019 | -1.7131 | 1.0000 | 14 | Tubulin alpha-1 chain |
| MSTRG.19252 | -3.3987 | 1.0000 | 15 | uncharacterized protein LOC100642617 isoform X3 |
| MSTRG.20047 | -3.0857 | 1.0000 | 16 | PAN2-PAN3 deadenylation complex catalytic subunit PAN2 isoform X3 |
| MSTRG.15344 | 1.8679 | 1.0000 | 17 | gag-pol polyprotein |
| MSTRG.15387 | -2.2987 | 1.0000 | 18 | ankyrin repeat and KH domain-containing protein 1 isoform X4 |
| MSTRG.17583 | -3.8835 | 1.0000 | 19 | NaN |
| MSTRG.4852 | 2.1813 | 1.0000 | 20 | membrane-associated protein Hem |
| MSTRG.8766 | 2.7234 | 1.0000 | 21 | balbiani ring protein 3-like |
| MSTRG.5796 | -1.3817 | 1.0000 | 22 | NaN |
| MSTRG.15482 | -1.8351 | 1.0000 | 23 | zinc finger SWIM domain-containing protein 8 isoform X1 |
| MSTRG.18933 | 0.7795 | 0.4627 | 24 | odorant receptor 46a-like |
| MSTRG.21015 | -3.5586 | 1.0000 | 25 | NaN |
| MSTRG.12442 | 3.6998 | 0.0759 | 26 | zinc finger protein YER130C isoform X2 |
| MSTRG.7733 | 0.8942 | 1.0000 | 27 | protein yippee-like 5 |
| MSTRG.9564 | 2.5118 | 1.0000 | 28 | uncharacterized protein LOC110119883 |
| MSTRG.21342 | 2.0539 | 1.0000 | 29 | NaN |
| MSTRG.8780 | 1.8734 | 1.0000 | 30 | uncharacterized protein LOC112212704 |
| MSTRG.10709 | -4.0532 | 1.0000 | 31 | NaN |
| MSTRG.14066 | -3.7618 | 1.0000 | 32 | 4-coumarate--CoA ligase 1-like |
| MSTRG.18133 | -2.4614 | 1.0000 | 33 | NaN |
| LOC105681602 | 1.3488 | 1.0000 | 34 | balbiani ring protein 3-like |
| MSTRG.20562 | -2.1733 | 1.0000 | 35 | RNA-directed DNA polymerase from mobile element jockey |
| MSTRG.2074 | 2.0966 | 1.0000 | 36 | NaN |
| MSTRG.2249 | -2.7250 | 1.0000 | 37 | putative uncharacterized protein DDB_G0277057 |
| MSTRG.18073 | -1.5528 | 1.0000 | 38 | E3 ubiquitin-protein ligase SIAH1-like |
| MSTRG.3573 | -2.7096 | 1.0000 | 39 | cartilage oligomeric matrix protein |
| MSTRG.11468 | 1.5309 | 1.0000 | 40 | ectonucleoside triphosphate diphosphohydrolase 5 isoform X2 |
| MSTRG.1825 | 1.4801 | 1.0000 | 41 | NaN |
| LOC105681186 | 2.5126 | 1.0000 | 42 | G-protein coupled receptor Mth2-like |
| MSTRG.15013 | 2.2747 | 1.0000 | 43 | NaN |
| MSTRG.11652 | -1.7649 | 1.0000 | 44 | NaN |
| MSTRG.9455 | -2.5856 | 1.0000 | 45 | replication protein A 70 kDa DNA-binding subunit |
| MSTRG.14915 | -3.1361 | 1.0000 | 46 | LIM/homeobox protein Lhx3 isoform X1 |
| MSTRG.7774 | -2.7167 | 1.0000 | 47 | NaN |
| MSTRG.4585 | -1.7744 | 1.0000 | 48 | receptor-type guanylate cyclase gcy-4-like isoform X2 |
| MSTRG.9773 | -1.0251 | 1.0000 | 49 | NaN |
| MSTRG.9630 | 1.1200 | 1.0000 | 50 | lysoplasmalogenase-like protein TMEM86A isoform X2 |
| MSTRG.3173 | 1.7870 | 0.8383 | 51 | cytochrome P450 9e2-like |
| MSTRG.13662 | -3.0904 | 1.0000 | 52 | proteasome activator complex subunit 4-like isoform X2 |
| MSTRG.16794 | 0.7455 | 1.0000 | 53 | NaN |
| MSTRG.21207 | -1.4786 | 1.0000 | 54 | serine/threonine-protein kinase SMG1 isoform X2 |
| MSTRG.18616 | -1.0445 | 1.0000 | 55 | NaN |
| MSTRG.4982 | -2.3372 | 1.0000 | 56 | transferrin |
| MSTRG.12519 | 3.3959 | 1.0000 | 57 | NaN |
| MSTRG.6801 | 1.5653 | 1.0000 | 58 | NaN |
| MSTRG.726 | -2.4791 | 1.0000 | 59 | NaN |
| MSTRG.18858 | 2.4604 | 1.0000 | 60 | NaN |
| MSTRG.18797 | -2.7311 | 1.0000 | 61 | coronin-1C-A |
| MSTRG.19964 | 3.2008 | 1.0000 | 62 | thyrotropin-releasing hormone-degrading ectoenzyme isoform X1 |
| MSTRG.2170 | -3.1263 | 1.0000 | 63 | hypothetical protein WH47_01332 |
| MSTRG.10517 | 3.5554 | 1.0000 | 64 | NaN |
| MSTRG.19220 | -3.3635 | 1.0000 | 65 | fatty acyl-CoA reductase wat-like |
| LOC100746040 | -3.2034 | 1.0000 | 66 | uncharacterized protein LOC100746040 |
| MSTRG.15012 | 3.9300 | 1.0000 | 67 | uncharacterized protein LOC100742276 |
| MSTRG.4831 | 1.0353 | 1.0000 | 68 | tachykinin-like peptides receptor 99D |
| MSTRG.14849 | 1.3630 | 1.0000 | 69 | NaN |
| MSTRG.9706 | 0.2902 | 1.0000 | 70 | gem-associated protein 2 |
| MSTRG.8508 | 0.5304 | 1.0000 | 71 | N-acetylgalactosaminyltransferase 6-like |
| MSTRG.21143 | -2.1638 | 1.0000 | 72 | carcinine transporter isoform X1 |
| MSTRG.3257 | -0.9088 | 1.0000 | 73 | Alpha-tocopherol transfer protein-like |
| MSTRG.21140 | -1.7618 | 1.0000 | 74 | NaN |
| MSTRG.21147 | -2.4463 | 1.0000 | 75 | 4-coumarate--CoA ligase 1 |
| MSTRG.5822 | -1.7236 | 1.0000 | 76 | NaN |
| MSTRG.21367 | 3.3807 | 1.0000 | 77 | glucose dehydrogenase [FAD, quinone]-like |
| MSTRG.16294 | 3.3230 | 1.0000 | 78 | band 7 protein AGAP004871 isoform X2 |
| MSTRG.15870 | -1.7701 | 1.0000 | 79 | (11Z)-hexadec-11-enoyl-CoA conjugase-like isoform X2 |
| MSTRG.8040 | 0.3449 | 1.0000 | 80 | pre-mRNA-splicing factor Syf2 |
| MSTRG.19005 | -3.6169 | 1.0000 | 81 | hypothetical protein WN51_02805 |
| MSTRG.13659 | -0.6371 | 1.0000 | 82 | proteasome activator complex subunit 4-like isoform X2 |
| MSTRG.10306 | -0.2700 | 1.0000 | 83 | ATP-dependent RNA helicase dbp2-like isoform X1 |
| MSTRG.4191 | -0.6581 | 1.0000 | 84 | NaN |
| MSTRG.1654 | -0.7423 | 1.0000 | 85 | sprT-like domain-containing protein Spartan |
| MSTRG.500 | 0.5563 | 1.0000 | 86 | tyrosine-protein phosphatase non-receptor type 1-like |
| MSTRG.2679 | -6.0332 | 1.0000 | 87 | NaN |
| MSTRG.20466 | -22.4594 | 0.0006 | 88 | NaN |
| MSTRG.10750 | -1.3781 | 1.0000 | 89 | hrp65 protein isoform X2 |
| MSTRG.18718 | -1.9180 | 1.0000 | 90 | WD repeat-containing protein 48 |
| MSTRG.14760 | 2.9057 | 1.0000 | 91 | uncharacterized protein LOC100743799 |
| MSTRG.15016 | 0.5677 | 1.0000 | 92 | 2-oxoglutarate-dependent dioxygenase htyE isoform X2 |
| MSTRG.19334 | -1.4883 | 1.0000 | 93 | NaN |
| MSTRG.11504 | 1.5113 | 1.0000 | 94 | NaN |
| MSTRG.13194 | 5.8735 | 0.8383 | 95 | uncharacterized protein LOC117237235 isoform X1 |
| MSTRG.2038 | -0.6181 | 1.0000 | 96 | NaN |
| MSTRG.7590 | 0.3070 | 1.0000 | 97 | transcription initiation factor TFIID subunit 6-like |
| MSTRG.6059 | -1.8860 | 1.0000 | 98 | Homogentisate 1,2-dioxygenase |
| MSTRG.15298 | 1.7787 | 1.0000 | 99 | NaN |
| MSTRG.14539 | 14.4897 | 0.1562 | 100 | NaN |
| MSTRG.3500 | -1.0535 | 1.0000 | 101 | uncharacterized protein LOC100747314 |
| MSTRG.18601 | -1.7324 | 1.0000 | 102 | synaptic vesicle membrane protein VAT-1 homolog-like |
| MSTRG.7659 | -0.3846 | 1.0000 | 103 | Protein disulfide-isomerase A6 |
| MSTRG.11065 | 1.2769 | 1.0000 | 104 | probable WRKY transcription factor protein 1 |
| MSTRG.18840 | -1.9928 | 1.0000 | 105 | ubiquitin carboxyl-terminal hydrolase 4-like isoform X3 |
| MSTRG.436 | 2.1747 | 1.0000 | 106 | forkhead box protein I1 |
| MSTRG.12434 | -1.0282 | 1.0000 | 107 | hypothetical protein WN48_10360 |
| MSTRG.9240 | -0.6025 | 1.0000 | 108 | hypoxia up-regulated protein 1 isoform X1 |
| MSTRG.12416 | -1.7447 | 1.0000 | 109 | tryptophan 2,3-dioxygenase |
| MSTRG.12777 | -1.1247 | 1.0000 | 110 | elongation of very long chain fatty acids protein AAEL008004 |
| MSTRG.19526 | 1.9357 | 1.0000 | 111 | GDP-D-glucose phosphorylase 1-like |
| MSTRG.12415 | -1.0023 | 1.0000 | 112 | tryptophan 2,3-dioxygenase |
| MSTRG.21242 | -1.1534 | 1.0000 | 113 | ATP-binding cassette sub-family G member 4 |
| MSTRG.362 | -20.8330 | 0.0030 | 114 | NaN |
| MSTRG.10890 | -2.0671 | 1.0000 | 115 | protein amnionless-like |
| LOC100744673 | -2.0474 | 1.0000 | 116 | green-sensitive opsin-like isoform X2 |
| MSTRG.11307 | 0.4356 | 1.0000 | 117 | methionine aminopeptidase 1 |
| MSTRG.487 | 0.9258 | 1.0000 | 118 | NaN |
| MSTRG.4977 | -1.7477 | 1.0000 | 119 | putative glutamate receptor |
| MSTRG.4784 | 0.2956 | 1.0000 | 120 | transcription factor IIIB 90 kDa subunit isoform X1 |
| MSTRG.8818 | -1.6297 | 1.0000 | 121 | uncharacterized protein LOC100740289 isoform X3 |
| MSTRG.4093 | 0.2892 | 1.0000 | 122 | E3 ubiquitin-protein ligase Bre1 isoform X2 |
| MSTRG.5949 | -2.1720 | 1.0000 | 123 | cell division cycle and apoptosis regulator protein 1-like |
| MSTRG.435 | 1.7094 | 1.0000 | 124 | NaN |
| MSTRG.12382 | 0.5350 | 1.0000 | 125 | Putative oxidoreductase GLYR1 like protein |
| MSTRG.10145 | -1.7454 | 1.0000 | 126 | uncharacterized protein LOC117162488 |
| MSTRG.19183 | 2.8764 | 1.0000 | 127 | patronin isoform X1 |
| MSTRG.4714 | -1.6354 | 1.0000 | 128 | Sorbitol dehydrogenase |
| MSTRG.209 | -2.2832 | 1.0000 | 129 | scavenger receptor class B member 1-like |
| MSTRG.12778 | -1.5820 | 1.0000 | 130 | elongation of very long chain fatty acids protein AAEL008004 |
| MSTRG.12847 | -1.6736 | 1.0000 | 131 | uncharacterized protein LOC100643622 isoform X3 |
| MSTRG.69 | 1.4398 | 1.0000 | 132 | uncharacterized protein LOC100744309 isoform X3 |
| MSTRG.360 | -1.4387 | 1.0000 | 133 | uncharacterized protein LOC117236781 isoform X2 |
| MSTRG.4262 | -6.6602 | 0.0818 | 134 | NaN |
| MSTRG.12190 | -2.0753 | 1.0000 | 135 | apolipophorin III-like protein |
| MSTRG.20782 | -1.3227 | 1.0000 | 136 | uncharacterized protein LOC110120041 |
| MSTRG.8423 | 0.4228 | 1.0000 | 137 | WD repeat domain-containing protein 83 |
| MSTRG.9880 | 1.9316 | 1.0000 | 138 | NaN |
| MSTRG.3256 | -2.3439 | 1.0000 | 139 | NaN |
| MSTRG.18758 | 4.3435 | 1.0000 | 140 | major royal jelly protein 1 |
| MSTRG.1056 | -2.3511 | 1.0000 | 141 | NaN |

**NaN = no available annotation.**

**Table S7**. Differentially expressed genes between infected sunflower- and uninfected sunflower-fed bees based on the DESeq2 model (FDR < 0.05). Gene descriptions based on top BLAST hits (E-value of 10^-25^) against all arthropod sequences in the NCBI non-redundant database.

| String Tie ID | Fold Change (log2) | FDR | Description |
| --- | --- | --- | --- |
| MSTRG.19477 | -29.5358 | 0.0000 | glucose dehydrogenase [FAD, quinone]-like |
| MSTRG.20478 | 24.3540 | 0.0004 | dynein beta chain, ciliary-like |
| MSTRG.20557 | 5.6167 | 0.0010 | NaN |
| MSTRG.21434 | 5.6302 | 0.0010 | NaN |
| MSTRG.11066 | 10.8113 | 0.0010 | probable endochitinase |
| MSTRG.9983 | -22.9533 | 0.0010 | uncharacterized protein LOC112212842 |
| MSTRG.20466 | 22.1348 | 0.0015 | NaN |
| MSTRG.21535 | 5.1220 | 0.0015 | hymenoptaecin |
| MSTRG.872 | -2.2853 | 0.0033 | piggyBac transposable element-derived protein 4-like |
| MSTRG.11147 | 5.9210 | 0.0046 | alkaline phosphatase 4-like |
| MSTRG.13521 | 12.0889 | 0.0167 | trypsin alpha-3-like |
| MSTRG.21576 | 10.6097 | 0.0479 | P-loop NTP hydrolase |

**NaN = no available annotation.**

**Table S8**. Top-ranked 80 genes that differentiate infected sunflower- and uninfected sunflower-fed bees based on an 80% correct classification rate using Machine Learning. A subset of genes from the DESeq2 model with an un-corrected p-value < 0.05 were ranked (Rank) based on information gain with respect to the treatment using the InfoGain attribute evaluator and Ranker search method in Weka. Gene descriptions based on top BLAST hits (E-value of 10^-25^) against all arthropod sequences in the NCBI non-redundant database.

| String Tie ID | Fold Change (log2) | FDR | Rank | Description |
| --- | --- | --- | --- | --- |
| MSTRG.10385 | -1.5601 | 1.0000 | 1 | NaN |
| MSTRG.13547 | 3.6600 | 1.0000 | 2 | NaN |
| MSTRG.357 | 1.7713 | 1.0000 | 3 | hypothetical protein WN51_13669 |
| MSTRG.2188 | 1.1836 | 1.0000 | 4 | E3 ubiquitin-protein ligase ZNF598 |
| MSTRG.10693 | 2.7794 | 0.9421 | 5 | Coiled-coil domain-containing protein 13 |
| MSTRG.4069 | 2.3744 | 1.0000 | 6 | angiotensin-converting enzyme-like |
| MSTRG.21015 | 2.9713 | 0.9409 | 7 | NaN |
| MSTRG.16171 | 1.2401 | 1.0000 | 8 | NaN |
| MSTRG.9133 | -1.6655 | 1.0000 | 9 | NaN |
| MSTRG.17084 | -1.4333 | 0.8988 | 10 | Probable RNaN-directed DNaN polymerase from transposon X-element |
| MSTRG.17231 | 0.9306 | 0.8868 | 11 | uridine diphosphate glucose pyrophosphatase |
| MSTRG.21291 | -1.0694 | 1.0000 | 12 | NaN |
| MSTRG.17222 | 1.3303 | 1.0000 | 13 | dolichyl-diphosphooligosaccharide--protein glycosyltransferase subunit STT3B isoform X1 |
| MSTRG.8981 | 1.1631 | 1.0000 | 14 | glycogen phosphorylase |
| MSTRG.16822 | -1.1778 | 1.0000 | 15 | NaN |
| MSTRG.13989 | 1.6588 | 1.0000 | 16 | leucine-rich repeat-containing protein 19 isoform X1 |
| MSTRG.7691 | 1.8210 | 1.0000 | 17 | NaN |
| MSTRG.12016 | 3.3784 | 0.2777 | 18 | maltase A2 isoform X1 |
| MSTRG.17560 | 2.2373 | 0.9201 | 19 | NaN |
| MSTRG.12597 | 1.3021 | 1.0000 | 20 | NaN |
| MSTRG.20336 | -1.5421 | 0.8868 | 21 | katanin p60 ATPase-containing subunit A1-like |
| MSTRG.3573 | 2.8266 | 1.0000 | 22 | cartilage oligomeric matrix protein |
| MSTRG.5135 | -0.9204 | 1.0000 | 23 | NaN |
| MSTRG.18933 | -0.5069 | 1.0000 | 24 | odorant receptor 46a-like |
| MSTRG.8881 | -2.3558 | 0.8868 | 25 | NaN |
| MSTRG.2074 | -3.3456 | 0.6639 | 26 | NaN |
| MSTRG.19080 | 1.0317 | 1.0000 | 27 | RNaN-binding protein 5-like isoform X1 |
| MSTRG.17603 | 3.4766 | 0.6639 | 28 | NaN |
| MSTRG.18344 | 1.8623 | 1.0000 | 29 | uncharacterized protein LOC117207620 |
| MSTRG.9723 | 2.3227 | 0.8988 | 30 | uncharacterized protein LOC100749322 isoform X2 |
| MSTRG.7554 | 1.3987 | 1.0000 | 31 | Low-density lipoprotein receptor-related protein 2 |
| MSTRG.14740 | -5.8033 | 0.9409 | 32 | NaN |
| MSTRG.4810 | 3.9253 | 1.0000 | 33 | NaN |
| MSTRG.707 | -1.4494 | 0.8868 | 34 | dynein heavy chain 8, axonemal |
| MSTRG.14903 | 5.0391 | 0.3783 | 35 | distal membrane-arm assembly complex protein 2 |
| MSTRG.388 | -1.1395 | 0.9895 | 36 | katanin p60 ATPase-containing subunit A1-like |
| MSTRG.20762 | -2.7651 | 0.8988 | 37 | uncharacterized protein LOC112212704 |
| MSTRG.502 | 1.2064 | 1.0000 | 38 | tyrosine-protein phosphatase non-receptor type 2 isoform X2 |
| MSTRG.3171 | -0.8400 | 1.0000 | 39 | NaN |
| MSTRG.8877 | 3.8289 | 1.0000 | 40 | NaNtterin-4-like isoform X1 |
| MSTRG.15843 | -3.1405 | 0.6639 | 41 | Transposon Tf2-9 polyprotein |
| MSTRG.20532 | 1.9928 | 0.9026 | 42 | polyubiquitin-B |
| MSTRG.7483 | 1.2665 | 1.0000 | 43 | RNaN-binding protein cabeza-like isoform X3 |
| MSTRG.19397 | -1.8033 | 1.0000 | 44 | alpha-tocopherol transfer protein-like |
| MSTRG.6121 | 1.7780 | 1.0000 | 45 | G-protein coupled receptor Mth2-like |
| MSTRG.18057 | 1.6273 | 1.0000 | 46 | NaN |
| MSTRG.8291 | -4.4958 | 0.3197 | 47 | protein hairy |
| MSTRG.4520 | 2.3034 | 1.0000 | 48 | protein FAM151B isoform X2 |
| MSTRG.13756 | -0.7557 | 1.0000 | 49 | titin isoform X4 |
| MSTRG.11824 | -1.3117 | 1.0000 | 50 | uncharacterized protein LOC117177545 |
| MSTRG.10505 | -0.9930 | 1.0000 | 51 | uncharacterized protein LOC100745137 |
| MSTRG.8298 | 1.3425 | 0.8868 | 52 | NaN |
| MSTRG.1008 | 5.0477 | 0.8825 | 53 | carbohydrate sulfotransferase 11-like |
| MSTRG.7122 | 3.1141 | 0.9501 | 54 | NaN |
| MSTRG.15344 | -2.7529 | 0.8825 | 55 | gag-pol polyprotein |
| MSTRG.4646 | 1.4957 | 1.0000 | 56 | NaN |
| MSTRG.20507 | 2.4118 | 1.0000 | 57 | uncharacterized protein LOC100750217 |
| MSTRG.7358 | -1.7270 | 0.9499 | 58 | uncharacterized protein LOC105680532 |
| MSTRG.6080 | 2.3889 | 1.0000 | 59 | protein obstructor-E-like |
| MSTRG.14101 | 4.6054 | 0.3706 | 60 | uncharacterized protein LOC117233211 isoform X8 |
| MSTRG.8779 | -3.0158 | 0.8868 | 61 | gag-pol polyprotein |
| MSTRG.21481 | -1.7375 | 0.8868 | 62 | Copia protein |
| MSTRG.15256 | 1.0433 | 1.0000 | 63 | NaN |
| MSTRG.9880 | -2.0864 | 1.0000 | 64 | NaN |
| MSTRG.10022 | -1.2107 | 1.0000 | 65 | NaN |
| MSTRG.21583 | 2.5511 | 1.0000 | 66 | reverse transcriptase |
| MSTRG.5446 | 1.7112 | 1.0000 | 67 | NaN |
| MSTRG.901 | -1.1467 | 1.0000 | 68 | hypothetical protein WN48_03057 |
| MSTRG.14495 | -1.8503 | 1.0000 | 69 | uncharacterized protein LOC112212704 |
| MSTRG.1182 | -1.2470 | 1.0000 | 70 | Ly6/PLAUR domain-containing protein 6B |
| MSTRG.4982 | 2.6530 | 1.0000 | 71 | transferrin |
| MSTRG.4114 | -1.9552 | 0.9992 | 72 | monocarboxylate transporter 12 isoform X1 |
| MSTRG.18601 | 1.5015 | 1.0000 | 73 | syNaNptic vesicle membrane protein VAT-1 homolog-like |
| MSTRG.5689 | 3.4669 | 1.0000 | 74 | NaN |
| MSTRG.7747 | 1.5130 | 1.0000 | 75 | uncharacterized protein LOC100740617 isoform X5 |
| MSTRG.11803 | 2.8612 | 1.0000 | 76 | glycine receptor subunit alpha-3-like isoform X1 |
| MSTRG.11112 | -1.9007 | 0.9477 | 77 | endocuticle structural glycoprotein SgAbd-4-like |
| MSTRG.2986 | -2.0885 | 0.8868 | 78 | hypothetical protein RF55_3628 |
| MSTRG.3431 | 2.4782 | 1.0000 | 79 | NaN |
| MSTRG.13494 | 4.4347 | 0.8965 | 80 | NaN |

**NaN = no available annotation.**

**Table S9**. Differentially expressed genes between infected wildflower- and uninfected wildflower-fed bees based on the DESeq2 model (FDR < 0.05). Gene descriptions based on top BLAST hits (E-value of 10^-25^) against all arthropod sequences in the NCBI non-redundant database.

| String Tie ID | Fold Change (log2) | FDR | Description |
| --- | --- | --- | --- |
| MSTRG.362 | -50.8282 | 0.0000 | NaN |
| MSTRG.11358 | -19.1894 | 0.0000 | glutamate receptor 3.2-like |
| MSTRG.7865 | 36.4587 | 0.0000 | ejaculatory bulb-specific protein 3-like |
| MSTRG.996 | -16.3274 | 0.0000 | NaN |
| MSTRG.15434 | 19.6215 | 0.0000 | NaN |
| LOC105680309 | -26.1608 | 0.0000 | probable ATP-dependent RNA helicase DDX46 |
| MSTRG.19495 | 17.7464 | 0.0000 | jerky protein homolog-like |
| MSTRG.12884 | -24.8345 | 0.0000 | NaN |
| MSTRG.8149 | -24.2671 | 0.0000 | uncharacterized protein LOC117204693 |
| MSTRG.9983 | 24.0341 | 0.0000 | uncharacterized protein LOC112212842 |
| MSTRG.14539 | 20.3251 | 0.0001 | NaN |
| MSTRG.19478 | -19.9946 | 0.0001 | glucose dehydrogenase [FAD, quinone]-like |
| MSTRG.18410 | -17.6068 | 0.0049 | NaN |
| MSTRG.19383 | -19.1270 | 0.0120 | digestive cysteine proteinase 1 |
| MSTRG.12886 | -18.5406 | 0.0219 | LOW QUALITY PROTEIN: uncharacterized protein LOC117236038 |
| LOC100747481 | -18.4691 | 0.0219 | LIM/homeobox protein Awh-like isoform X1 |
| MSTRG.15744 | 16.6987 | 0.0267 | uncharacterized protein LOC112213491 |

**NaN = no available annotation.**

**Table S10**. Top-ranked 98 genes that differentiate infected wildflower- and uninfected wildflower-fed bees based on an 100% correct classification rate using Machine Learning. A subset of genes from the DESeq2 model with an un-corrected p-value < 0.05 were ranked (Rank) based on information gain with respect to the treatment using the InfoGain attribute evaluator and Ranker search method in Weka. Gene descriptions based on top BLAST hits (E-value of 10^-25^) against all arthropod sequences in the NCBI non-redundant database.

| String Tie ID | Fold Change (log2) | FDR | Rank | Description |
| --- | --- | --- | --- | --- |
| MSTRG.2970 | -0.9920 | 1.0000 | 1 | striatin-interacting protein 1 |
| MSTRG.4529 | 1.0832 | 1.0000 | 2 | NaN |
| MSTRG.6215 | 1.0238 | 1.0000 | 3 | lachesin-like isoform X2 |
| MSTRG.2011 | 1.3924 | 1.0000 | 4 | protein BTG2-like |
| MSTRG.20954 | 0.6861 | 1.0000 | 5 | uncharacterized protein LOC117157497 isoform X2 |
| MSTRG.8475 | 1.3709 | 1.0000 | 6 | proclotting enzyme-like |
| MSTRG.19449 | 1.1841 | 1.0000 | 7 | NaN |
| MSTRG.12964 | 0.5895 | 1.0000 | 8 | NaN |
| MSTRG.15486 | -2.2736 | 0.7421 | 9 | alanine--glyoxylate aminotransferase 2-like |
| MSTRG.15384 | -0.9837 | 1.0000 | 10 | NaN |
| MSTRG.3256 | -1.9381 | 1.0000 | 11 | NaN |
| MSTRG.6710 | -1.5980 | 1.0000 | 12 | rho GTPase-activating protein 44 isoform X3 |
| MSTRG.298 | 3.7106 | 1.0000 | 13 | RNA-binding protein squid |
| MSTRG.16875 | 1.6594 | 1.0000 | 14 | elongation of very long chain fatty acids protein 6-like |
| MSTRG.4708 | 1.0848 | 0.8668 | 15 | anoctamin-4 isoform X1 |
| MSTRG.10982 | 1.5617 | 1.0000 | 16 | NaN |
| MSTRG.3512 | -2.6495 | 1.0000 | 17 | NaN |
| MSTRG.18718 | -3.3143 | 1.0000 | 18 | WD repeat-containing protein 48 |
| MSTRG.19948 | -2.1623 | 1.0000 | 19 | SH3 domain-binding protein 5 homolog |
| MSTRG.12451 | 0.8816 | 1.0000 | 20 | uncharacterized protein LOC117162246 |
| MSTRG.6794 | -1.1977 | 1.0000 | 21 | NaN |
| MSTRG.6618 | 1.1992 | 1.0000 | 22 | transposase |
| MSTRG.9655 | 1.0977 | 1.0000 | 23 | NaN |
| MSTRG.8015 | -1.8438 | 1.0000 | 24 | ras-related GTP-binding protein A |
| LOC100740223 | 2.2893 | 1.0000 | 25 | homeobox protein B-H2-like |
| MSTRG.695 | 2.7745 | 1.0000 | 26 | NaN |
| MSTRG.5529 | -1.3283 | 1.0000 | 27 | uncharacterized protein LOC102671994 |
| MSTRG.5302 | -2.6243 | 1.0000 | 28 | 40S ribosomal protein S17 |
| MSTRG.14346 | 0.7129 | 1.0000 | 29 | ATP-binding cassette sub-family G member 4-like isoform X3 |
| MSTRG.12432 | 1.1147 | 1.0000 | 30 | Zinc finger protein Xfin |
| MSTRG.16211 | -0.8547 | 1.0000 | 31 | NaN |
| MSTRG.2498 | -2.5501 | 1.0000 | 32 | HAUS augmin-like complex subunit 3 |
| MSTRG.7174 | -2.7355 | 1.0000 | 33 | NaN |
| MSTRG.8538 | -1.3196 | 1.0000 | 34 | NPC intracellular cholesterol transporter 2-like |
| MSTRG.16762 | 1.4006 | 1.0000 | 35 | hexamerin-like |
| MSTRG.4948 | -1.0546 | 1.0000 | 36 | Pupal cuticle protein C1B |
| MSTRG.3866 | -2.0170 | 1.0000 | 37 | dnaJ homolog subfamily C member 11 |
| MSTRG.15012 | 4.0038 | 1.0000 | 38 | uncharacterized protein LOC100742276 |
| MSTRG.2919 | -1.5749 | 1.0000 | 39 | mitochondrial import receptor subunit TOM20 homolog |
| MSTRG.8766 | 4.1261 | 0.7299 | 40 | balbiani ring protein 3-like |
| LOC105681602 | -1.5017 | 1.0000 | 41 | balbiani ring protein 3-like |
| MSTRG.1111 | -2.5279 | 1.0000 | 42 | NaN |
| MSTRG.12405 | -2.1173 | 1.0000 | 43 | multidrug resistance protein homolog 49 isoform X1 |
| MSTRG.17808 | -1.3422 | 1.0000 | 44 | asparagine--tRNA ligase, cytoplasmic |
| MSTRG.13109 | -1.0957 | 1.0000 | 45 | DDB1- and CUL4-associated factor 12 |
| MSTRG.14625 | -2.2510 | 1.0000 | 46 | NaN |
| MSTRG.6962 | 2.8354 | 1.0000 | 47 | NaN |
| MSTRG.7220 | -3.9030 | 1.0000 | 48 | serine/threonine-protein phosphatase 1 regulatory subunit GAC1-like |
| MSTRG.19964 | 4.2505 | 1.0000 | 49 | thyrotropin-releasing hormone-degrading ectoenzyme isoform X1 |
| MSTRG.19027 | 1.5203 | 1.0000 | 50 | uncharacterized protein C553.10-like, partial |
| MSTRG.13943 | -1.3350 | 1.0000 | 51 | reverse transcriptase |
| MSTRG.10598 | -1.7071 | 1.0000 | 52 | TBC1 domain family member 25 |
| MSTRG.15604 | -3.4310 | 1.0000 | 53 | uncharacterized protein LOC100746190 |
| MSTRG.20722 | 1.1636 | 1.0000 | 54 | Retrovirus-related Pol polyprotein from transposon TNT 1-94 |
| MSTRG.119 | 1.5195 | 1.0000 | 55 | conserved hypothetical protein |
| MSTRG.14073 | -2.7443 | 1.0000 | 56 | luciferin 4-monooxygenase-like isoform X6 |
| MSTRG.10435 | -1.5915 | 1.0000 | 57 | NaN |
| MSTRG.1380 | -2.2248 | 1.0000 | 58 | NaN |
| MSTRG.11539 | -2.6621 | 1.0000 | 59 | nuclear fragile X mental retardation-interacting protein 1-like |
| MSTRG.7048 | 2.0188 | 1.0000 | 60 | piggyBac transposable element-derived protein 4-like |
| MSTRG.8362 | -3.5896 | 1.0000 | 61 | tRNA modification GTPase GTPBP3, mitochondrial isoform X2 |
| LOC112213846 | -3.4330 | 1.0000 | 62 | Protein lozenge |
| MSTRG.20983 | 5.7743 | 1.0000 | 63 | NaN |
| MSTRG.8229 | 4.3588 | 1.0000 | 64 | hypothetical protein WN48_05001 |
| MSTRG.13254 | 1.3363 | 1.0000 | 65 | zinc finger CCHC domain-containing protein 24-like |
| LOC100747736 | 1.6070 | 1.0000 | 66 | circadian locomoter output cycles protein kaput |
| MSTRG.14055 | 2.4815 | 1.0000 | 67 | NaN |
| MSTRG.15013 | 1.9031 | 1.0000 | 68 | NaN |
| MSTRG.4355 | -0.7193 | 1.0000 | 69 | inhibitor of nuclear factor kappa-B kinase subunit epsilon |
| MSTRG.15018 | -0.2317 | 1.0000 | 70 | translation initiation factor IF-2, mitochondrial |
| MSTRG.14908 | -1.5226 | 1.0000 | 71 | NaN |
| LOC100747481 | -18.4691 | 0.0219 | 72 | LIM/homeobox protein Awh-like isoform X1 |
| MSTRG.20614 | -2.6889 | 1.0000 | 73 | mitochondrial potassium channel ATP-binding subunit |
| MSTRG.9393 | 1.3862 | 1.0000 | 74 | NaN |
| MSTRG.8499 | -0.3870 | 1.0000 | 75 | growth hormone-regulated TBC protein 1 |
| MSTRG.4751 | -0.7321 | 1.0000 | 76 | COP9 signalosome complex subunit 4 |
| MSTRG.2588 | 1.7677 | 1.0000 | 77 | NaN |
| MSTRG.3654 | 0.7925 | 1.0000 | 78 | PREDICTED: uncharacterized protein LOC105662916 |
| MSTRG.8149 | -24.2671 | 0.0000 | 79 | uncharacterized protein LOC117204693 |
| MSTRG.6434 | 0.9958 | 1.0000 | 80 | ribose-phosphate pyrophosphokinase 1 isoform X2 |
| MSTRG.16362 | -0.6239 | 1.0000 | 81 | putative inorganic phosphate cotransporter |
| MSTRG.1744 | 0.9534 | 1.0000 | 82 | NaN |
| MSTRG.1112 | 3.0768 | 1.0000 | 83 | cytochrome P450 307a1-like |
| MSTRG.8141 | 1.1538 | 1.0000 | 84 | NaN |
| MSTRG.10228 | 2.8567 | 1.0000 | 85 | NaN |
| MSTRG.2597 | -0.2316 | 1.0000 | 86 | conserved oligomeric Golgi complex subunit 5 |
| MSTRG.19478 | -19.9946 | 0.0001 | 87 | glucose dehydrogenase [FAD, quinone]-like |
| MSTRG.435 | 1.7020 | 1.0000 | 88 | NaN |
| MSTRG.2368 | -0.8815 | 1.0000 | 89 | NaN |
| MSTRG.15256 | -0.9675 | 1.0000 | 90 | NaN |
| MSTRG.21259 | 2.0608 | 1.0000 | 91 | putative uncharacterized protein DDB_G0289041 |
| MSTRG.6137 | 0.9310 | 1.0000 | 92 | aquaporin AQPAn.G isoform X3 |
| MSTRG.2534 | -1.2250 | 1.0000 | 93 | RNA-directed DNA polymerase from mobile element jockey-like |
| MSTRG.16044 | -2.4720 | 1.0000 | 94 | uncharacterized protein LOC105681292 |
| MSTRG.1526 | 1.8967 | 1.0000 | 95 | NaN |
| MSTRG.2166 | -1.2922 | 1.0000 | 96 | uncharacterized protein LOC100740206 |
| MSTRG.10306 | -0.3938 | 1.0000 | 97 | ATP-dependent RNA helicase dbp2-like isoform X1 |
| MSTRG.868 | 1.5115 | 1.0000 | 98 | Mitogen-activated protein kinase kinase kinase 15 |

**NaN = no available annotation.**

**Table S11**. Final sample sizes for **Timing of sunflower pollen effect** pilot experiment. Numeric values indicate the number of bees that consumed a net positive amount of pollen per each pollen type (sunflower or wildflower) per each timing treatment (24H, 48H, 72H, 96H and 168H).

| Pollen type | 24H | 48H | 72H | 96H | 168H |
| --- | --- | --- | --- | --- | --- |
| Sunflower | 5 | 7 | 10 | 10 | 13 |
| Wildflower | 5 | 4 | 10 | 10 | 13 |

**References**

1. LoCascio GM, Pasquale R, Amponsah E, Irwin RE, Adler LS. Effect of timing and exposure of sunflower pollen on a common gut pathogen of bumble bees. Ecological Entomology. 2019;44(5):702–10.

2. Kearns CA, Inouye DW. Techniques for pollination biologists. Niwot, CO: University Press of Colorado; 1993.

3. Blackmore S, Wortley AH, Skvarla JJ, Rowley JR. Pollen wall development in flowering plants. New Phytologist. 2007 May 1;174(3):483–98.

4. Richardson LL, Adler LS, Leonard AS, Andicoechea J, Regan KH, Anthony WE, et al. Secondary metabolites in floral nectar reduce parasite infections in bumblebees. Proceedings of the Royal Society B. 2015 Mar 22;282(1803):20142471.

5. Giacomini JJ, Connon SJ, Marulanda D, Adler LS, Irwin RE. The costs and benefits of sunflower pollen diet on bumble bee colony disease and health. Ecosphere. 2021;00(00):e03663.

6. Giacomini JJ, Leslie J, Tarpy DR, Palmer-Young EC, Irwin RE, Adler LS. Medicinal value of sunflower pollen against bee pathogens. Scientific Reports. 2018;8(1):14394.

7. Manson JS, Otterstatter MC, Thomson JD. Consumption of a nectar alkaloid reduces pathogen load in bumble bees. Oecologia. 2010 Jan 1;162(1):81–9.

8. Nooten SS, Rehan SM. Historical changes in bumble bee body size and range shift of declining species. Biodiversity and Conservation. 2020;29(2):451–67.

9. R Core Team. R: A Language and Environment for Statistical Computing [Internet]. Vienna, Austria: R Foundation for Statistical Computing; 2020. Available from: https://www.R-project.org/

10. Venables WN, Ripley BD. Modern Applied Statistics with S [Internet]. Fourth. New York: Springer; 2002. Available from: http://www.stats.ox.ac.uk/pub/MASS4/

11. Lenth R. emmeans: Estimated Marginal Means, aka Least-Squares Means. [Internet]. 2020. Available from: https://CRAN.R-project.org/package=emmeans
